# Theoretical Study of an Authentic Hydrocarbon Ion Pair

**DOI:** 10.1021/acsomega.4c04914

**Published:** 2024-08-02

**Authors:** Elizete Ventura, Gessenildo Pereira Rodrigues, Ezequiel Fragoso Vieira Leitão, Silmar Andrade do Monte

**Affiliations:** † Departamento de Química, CCEN, Universidade Federal da Paraíba, 58059-900 João Pessoa, Brazil; ‡ Unidade Acadêmica de Ciências Exatas e da Natureza, Universidade Federal de Campina Grande, Cajazeiras, PB 58900-000, Brazil

## Abstract

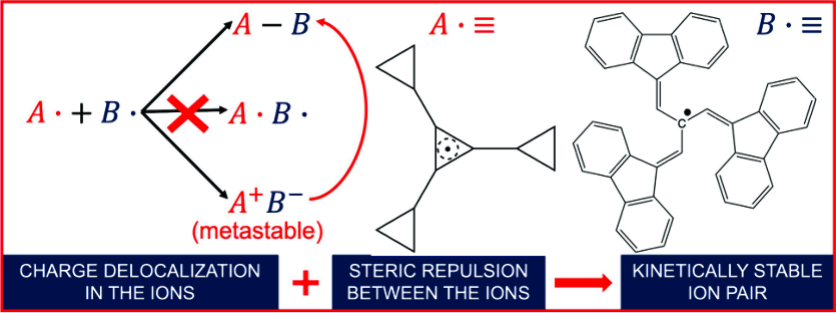

For many years researchers believed that hydrocarbons only contain covalent bonds. However, since 1985 Okamoto et al. demonstrated the formation of hydrocarbon salts in several systems, demolishing the structural principle that hydrocarbons only contain covalent bonds. Despite the great importance of this outcome to the study of chemical bonds, quantum chemical calculations on these systems are essentially nonexistent. The stability of the hydrocarbon ions along with the steric hindrance associated with the formation of the covalent bond contribute to their occurrence either in solution (dissociated) or in the solid state. These facts along with the common formation of ion pairs in solvents of low polarity motivated us to search for hydrocarbon ion pairs in the gas phase. Its energetics has also been studied in four nonprotic solvents, through a continuum solvation model (CPCM). DFT and CASSCF calculations indicate a metastable and highly polar ion pair between the tricyclopropylcyclopropenylium cation and a simplified Kuhn’s anion. The barrier to the covalent structure varies from ∼4.8 to 14.4 kcal/mol, while the energy difference between the ion pair and the covalent form varies from ∼4.3 to 25.4 kcal/mol. The obtained theoretical results along with previous experimental results suggest the following strategy to obtain kinetically and thermodynamically stable hydrocarbon ion pairs: choose very stable hydrocarbon ions and systematically increase the steric hindrance between them.

## Introduction

In 1985 Okamoto et al.^1^ demonstrated, for the first time, the formation of a genuine hydrocarbon salt. Such salt is formed by the Agranat’s cation and the Kuhn’s anion and it is remarkably stable under Ar in the dark at 10 °C.^1^ After this seminal work, several other hydrocarbon salts have been prepared and characterized.^2−5^ For instance, in ref.^2^ the salt is formed by combining the tricyclopropylcyclopropenylium cation and the Kuhn’s anion. The common features of these hydrocarbon salts are (i) They contain highly stable ions; (ii) There is a large steric hindrance between the opposite ions.

The existence of the mentioned salts is very important to the study of chemical bonds, as it has demolished the structural principle that genuine hydrocarbons only contain covalent bonds. Nevertheless, quantum chemical calculations on these systems are virtually nonexistent, probably due to their large sizes. To the best of our knowledge, there is only one work, by Fujimoto et al., in which hydrocarbon ion pairs of very small model systems (as, for instance, the methyl anion interacting with the cyclopropenium cation) are studied through a very simple frontier orbitals model.^6^

**Figure 1:**
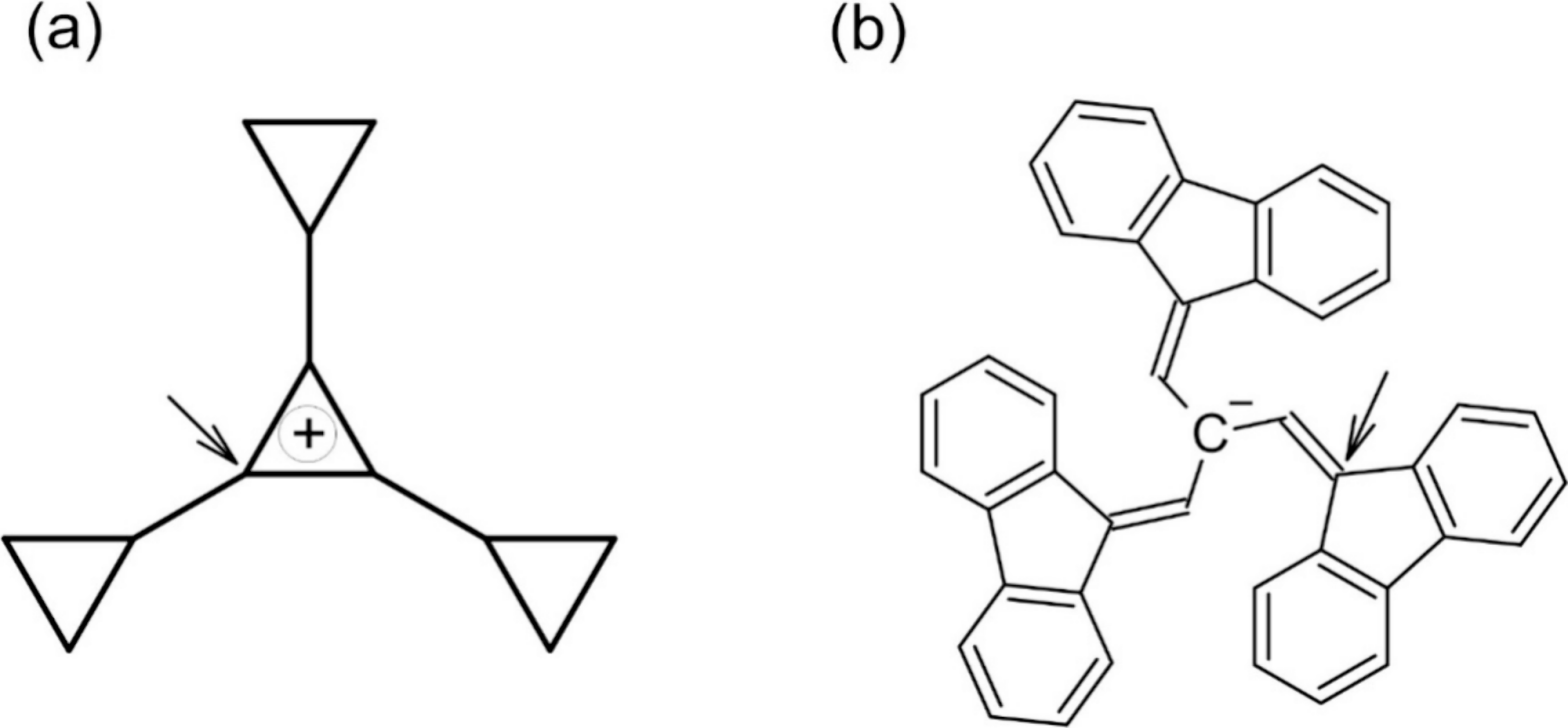
Ions forming the ion pair studied in this work: (a) tricyclopropylcyclopropenylium cation; (b) simplified Kuhn’s anion. The complete Kuhn’s anion is shown in ref (2). The arrows indicate the atoms that form the C–C bond in the covalent form.

In this work, we study a much more realistic hydrocarbon ion pair, the one formed between the tricyclopropylcyclopropenylium cation and a slightly simplified Kuhn’s anion. The cation is the same as in ref (2), while some of the benzene rings of Kuhn’s anion^2^ have been removed (see Figure 1). This is the first realistic hydrocarbon ion pair studied using quantum chemistry methods with expected good accuracy. DFT and CASSCF calculations are employed. The barrier from the ion pair to the covalent form varies from ∼4.8 to 14.4 kcal/mol, depending on the computational level and on the solvent. On the other hand, the energy difference between the ion pair and the covalent form varies from ∼4.3 to 25.4 kcal/mol. Our results indicate that, even in the gas phase, the ion pair has a significant kinetic stability, and it is only 25.4 kcal/mol less stable than the covalent form, a remarkable result for a hydrocarbon. As the polarity of the solvent increases, the kinetic stability as well as the thermodynamic stability of the ion pair tend to increase. The ion pair is characterized by its dipole moment, electrostatic potential, NBO charges, structure, occupation numbers of the natural orbitals, vertical excitation energy to S_1,_ and singlet–triplet gap. Its possible formation from the corresponding radicals, followed by its identification, in helium nanodroplets (HND), is also discussed.

## Computational Details

The ions forming the ion pair studied in this work are shown in Figure 1. First, the geometries of the ion pair, the covalent form, and the transition state (TS) connecting the previous structures are fully optimized at the DFT level using the M05-2X functional^7^ with the mixed 6-31+G*(C)/sto-3G(H) basis set.^8−11^ This basis set has been used for all DFT calculations. Frequency calculations have been performed to confirm that the ion pair and the covalent form are minima while the TS is a first-order saddle point that connects the two minima. The M05-2X functional^7^ has been chosen based on its very good performance for the calculation of ion-pair binding energies of pyrrolidinium-based ionic liquids^12^ and on previous studies with systems containing aromatic carbocations.^13,14^

The next step consists of a relaxed scan at the M05-2X level (using the same basis set as before) along the C–C bond formed by the C atoms indicated in Figure 1. According to ref.^2^ this is the covalent bond formed when the ions react to form the covalent structure (see scheme 1 of ref.^2^). The relaxed scan is done in both directions, that is, from the TS to the ion pair and from the TS to the covalent form. The corresponding steps are 0.308 and 0.217 Å, respectively. The first step (0.308 Å) corresponds to the difference between the C–C distance in the ion pair (3.669 Å) and in the TS (2.434 Å) divided by four. Similarly, the second step (0.217 Å) corresponds to the difference between the C–C distance in the ion TS (2.434 Å) and in the covalent form (1.565 Å) divided by four. Three intermediate structures are obtained in both directions. All nine structures (the ion pair, TS, covalent form, and the six intermediate structures) are then used to perform single-point calculations at the CASSCF level, with six electrons distributed in six active orbitals. This choice for the active space is based on test calculations performed with other active spaces, as explained later. A mixed cc-pVDZ(C)/sto-3G(H) basis set^11,15^ has been used for these calculations.

The first set of guess orbitals to be used at the CASSCF level, for the TS structure, has been generated at the RHF/sto-3G level. The HOMO and LUMO+2 orbitals are the σ and σ* orbitals of the C–C bond, respectively, both mixed with π orbitals. Three of the remaining four guess orbitals (HOMO–1, HOMO–2, and LUMO+1) are π orbitals mainly located at the anionic fragment (see Figure 1), while the LUMO is located at both fragments. This set of orbitals is then used as guess orbitals for the CAS(6,6) calculation with the cc-pVDZ(C)/sto-3G(H) basis set. In the final set of converged active orbitals for the TS structure, the σ and σ* orbitals (again mixed with π orbitals) correspond to the HOMO–1 and LUMO+1, respectively, while the remaining four active orbitals are π orbitals mainly located at the anionic fragment. The first part of the relaxed scan is in the direction of the ion pair structure, that is, the C–C bond distance is increased in equal steps of 0.308 Å, as explained before. The whole set of converged orbitals obtained for TS is then used for the next point, and so on until the ion pair structure is achieved. In the second part of the relaxed scan the C–C bond distance is decreased in equal steps of 0.217 Å. Again, the whole set of converged orbitals of TS is used for the next point, and so on, until the covalent structure is achieved. The converged active orbitals obtained for all structures of the relaxed scan are shown in Figure S1 in the Supporting Information (SI). The natural orbitals of all these structures were also computed (at the CASSCF level), and the occupation number of the lowest unoccupied natural orbital (LUNO) was used to infer the diradical character of S_0_ along the scan.

To analyze the diradical character of the ion pair its lowest-lying triplet state is also computed at the M05-2X and CASSCF levels. In the case of the latter level the singlet orbitals obtained at the same level are used as the guess orbitals, both with the cc-pVDZ(C)/sto-3G(H) basis set. The converged active orbitals obtained for the triplet state are shown in Figure S2 in the SI. An additional analysis of the diradical character has been obtained through a comparison between restricted and unrestricted DFT (UDFT) calculations.

From the structural point of view, the cationic fragment of the ion pair is compared to the free (optimized) cation and to its reduced (that is, radical) form, through the root-mean-square deviation (RMSD) parameter. Similarly, the anionic fragment is compared to the free (optimized) anion and its oxidized form. These comparisons are carried out with the chemcraft software.^16^ RMSD corresponds to the average distance between the atoms of two superimposed structures. The maximum superposition is achieved rotating and translating one of the two structures to minimize the RMSD value. The geometry optimizations of the radicals have been performed at the restricted open-shell formalism at the DFT level, RODFT.^17^

Finally, two additional properties have also been used to characterize the ion pair, that is, the NBO charges of the fragments and the vertical excitation energy to S_1_, at the M05-2X and CASSCF levels. In the case of the CASSCF calculations, the converged orbitals of S_0_ are used as guess orbitals for S_1_. The converged active orbitals obtained for the S_1_ state are shown in Figure S3 in the SI. These excited state calculations have also been performed with the cc-pVDZ(C)/sto-3G(H) basis set. The NBO charge partitioning scheme has been chosen due to its relatively low basis set dependence.^18^

The basis set superposition error (BSSE) has been considered for the ion pair and TS, through the counterpoise (CP) method^19^ included in the geometry optimizations and frequency calculations^20^ carried out at the DFT level. Results at the CASSCF level including the CP method have not been included due to convergence problems. However, CASSCF calculations have also been performed using the CP-corrected geometries. Most of the calculations have been performed with the Gaussian 09 software using its default setup.^21^ The only exception is the UDFT calculation, for which the option guess = (mix,always) has been employed. In this case, this option has been used due to the possible open-shell nature of the singlet state of the ion pair.

The solvent effects have also been taken into account at the DFT level, by using the continuum solvation model CPCM,^22,23^ as implemented in the Gaussian 09 software.^21^ The following four nonprotic solvents of increasing dielectric constant have been considered in this study: THF, dichloromethane, dichloroethane (EDC) and acetonitrile. These are the same solvents whose ionic hydrocarbon solutions have been studied in ref.^3^

In order to characterize the interaction between the ions in the ion pair an energy decomposition analysis (EDA) calculation has also been carried out, at the DFT level in gas phase, according to the decomposition scheme suggested by Su and Li.^24^ This calculation has been performed by using the GAMESS software.^25^

## Results and Discussion

### Gas Phase

**Figure 2:**
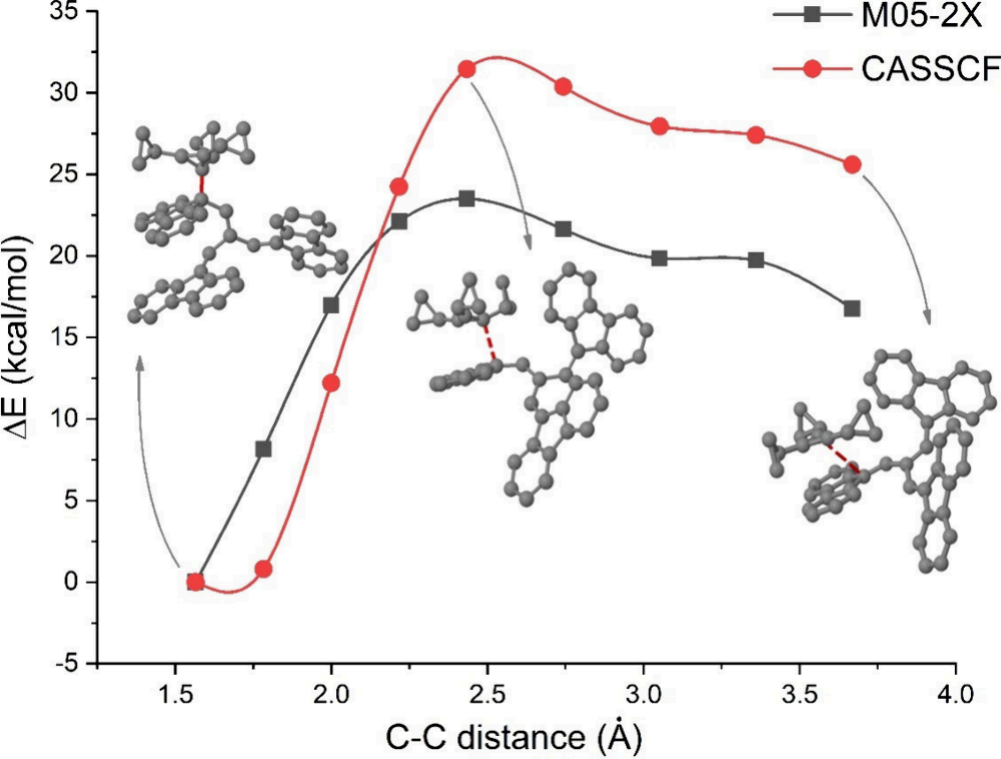
Structures of the covalent form, TS, and ion pair (from left to right) as well as relaxed scan obtained at the M05-2X/6-31+G*(C)/sto-3G(H) level. CASSCF energies of S_0_, computed with the cc-pVDZ(C)/sto-3G(H) basis sets, are also shown. The H atoms have been omitted for clarity. The two nonbonded C atoms connected by a dotted line, in the ion pair and TS, indicate the C atoms that form the covalent bond between the fragments, and which define the scan coordinate (see also Figure 1). The values are relative to the energy of the covalent form.

The structures of the ion-pair, TS, and the covalent form, obtained at the DFT level, are shown in Figure 2. The DFT and CASSCF energies of S_0_ along the C–C distance between the atoms indicated in Figure 1 are also given. The distance between these atoms in the ion pair, TS, and covalent structure are 3.669, 2.434, and 1.565 Å, respectively. Including the CP correction the first two distances change slightly to 3.696 and 2.430 Å. It is important to point out that the CP calculations require integer charges for the fragments. However, as discussed later, at the TS the computed charges deviate considerably from integer values. Therefore, the CP calculations of TS should be taken with caution. The distance between the centers of masses of the ions, in the ion pair, is 5.085 Å, much larger than that between the C atoms which form the covalent bond. The corresponding distance for the CP corrected structure is significantly larger, 5.244 Å. The Cartesian coordinates of the three optimized structures are given in Table S1 in the SI.

**Figure 3:**
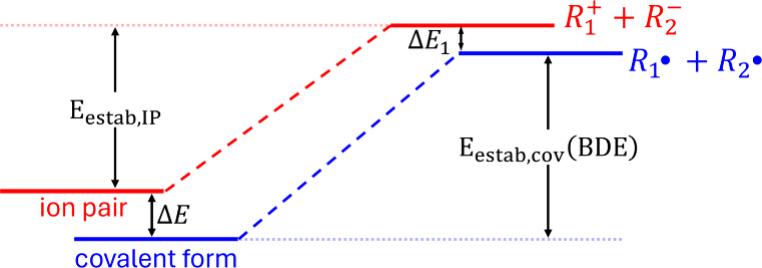
Scheme showing the stabilization energies of the ion pair and the covalent form (*E*_estab,IP_ and *E*_estab,cov_), the energy differences between the two dissociation channels (Δ*E*_1_) and the energy difference between the ion pair and the covalent form (Δ*E*).

The ionic channel (R_1_^+^ + R_2_^–^, where R_1_^+^ and R_2_^–^ are the ions shown in Figure 1) is 25.58 kcal/mol (Δ*E*_1_, see Figure 3) larger than the neutral channel (R_1_^•^ + R_2_^•^), at the M05-2X level. Despite the larger stabilization energy due to the formation of the ion-pair (E_estab,IP_), 70.90 kcal/mol, as compared to that associated with formation of the covalent form (*E*_estab,conv_), 61.90 kcal/mol, the ion pair is 16.58 kcal/mol (Δ*E*) less stable than the covalent form, in the gas phase. The reason is that the difference of 9.0 kcal/mol between the two stabilization energies is not enough to overcome the large energy difference between the two channels. It is important to point out that the value of 61.90 kcal/mol corresponds to the bond dissociation energy (at 0K) of the studied covalent (C–C) bond. This value is considerably lower than that for C–C bonds in alkanes which do not contain significant steric repulsions, ∼87–90 kcal/mol.^26^ However, as such type of repulsion increases, as when a C–C bond is formed between two t-butyl radicals, the value decreases to ∼78 kcal/mol, which is in line with the result obtained for the covalent form of the system formed between the cyclopropenyl radical and that corresponding to the oxidized form of the simplified Kuhn’s anion shown in Figure 1(b). The bond dissociation energy of this covalent form is 3.4 kcal/mol larger than the covalent form shown in Figure 2, which shows the weakening of the C–C bond due to the steric repulsion of the cyclopropyl substituents. Another very important outcome due to these substituents is the occurrence of the ion pair structure (see Figure 2), that is, if they are replaced by H atoms only the covalent form is obtained, as discussed later.

All these values have been computed including the zero-point energies (ZPE). The 6-31+G*(C)/sto-3G(H) basis set has been used for the geometry optimizations of the three radicals and for the ZPE calculation of the tricyclopropylcyclopropenyl (see Figure 1(a)) and cyclopropenyl radicals. In the case of the ZPE calculation for the larger radical (see Figure 1(b)) the 3-21G*(C)/sto-3G(H) basis set has been used, due to the large computational cost of the frequency calculation of the larger radical. Its use is justified by test calculations for the tricyclopropylcyclopropenyl and cyclopropenyl radicals, which lead to a difference of at most only 1.00 kcal/mol (for the tricyclopropylcyclopropenyl radical) between the ZPE values computed with the 3-21G*(C)/sto-3G(H) and the 6-31+G*(C)/sto-3G(H) basis sets.

At the DFT level, the barrier and the reaction energy (Δ*E*) for the ion pair → covalent form reaction (see Figure 2) are 5.71 and −16.58 kcal/mol, respectively (both computed including the ZPE). Using the CASSCF energies (with the cc-pVDZ(C)/sto-3G(H) basis set) and the ZPE computed at the M05-2X level, one obtains a decrease of only 0.89 kcal/mol for the barrier. On the other hand, the decrease for Δ*E* is much more pronounced, 8.84 kcal/mol. In order to check the reliability of the 6-31+G*(C)/sto-3G(H) basis set, single-point calculations have been performed for the three stationary points shown in Figure 2, with the 6-31+G* basis set at the M05-2X level. For this new basis set, Δ*E* increases ∼1.6 kcal/mol, while the barrier increases only ∼0.5 kcal/mol. Thus, the 6-31+G*(C)/sto-3G(H) basis set is a good approximation for the 6-31+G* basis set, at least regarding these two energy differences.

As mentioned earlier, the configurations of the CASSCF calculations have been generated from six active orbitals and electrons. MCSCF calculations can be very difficult to converge, especially for systems without symmetry. This is exactly the case for the structures studied in this work. Although larger active spaces are computationally feasible, CAS(6,6) was the largest active space leading to a computationally feasible calculation which also simultaneously satisfied the following two criteria: (i) convergence has been achieved along all points of the relaxed scan (always using the set of converged orbitals of the previous point, as explained before); and (ii) a smooth potential energy curve has been obtained (see Figure 2). It is also important to mention that for molecules with large π systems one does not necessarily need to use large active spaces in order to obtain reliable results.^27^ However, a comparison between our results and those obtained with larger active spaces is very welcome.

With the inclusion of the CP correction at the M05-2X level the barrier increases very slightly, from 5.71 to 6.04 kcal/mol. However, Δ*E* decreases significantly, from −16.58 to −20.72 kcal/mol. The almost negligible change for the barrier can be explained by a very similar CP correction for both ion pair and TS. Conversely, the change in Δ*E* is only due to the CP correction of the ion pair, which explains the larger effect.

It is worth noting that at the CASSCF level one uses the CP corrected ZPE and geometries obtained at the M05-2X level. However, the CASSCF energies do not include the CP correction (due to convergence problems, as mentioned before). Thus, the CP correction at this level can be considered incomplete, leading to almost negligible changes for the barrier (from 4.82 to 4.92 kcal/mol) and for Δ*E* (from 25.42 to 24.52 kcal/mol).

**Figure 4:**
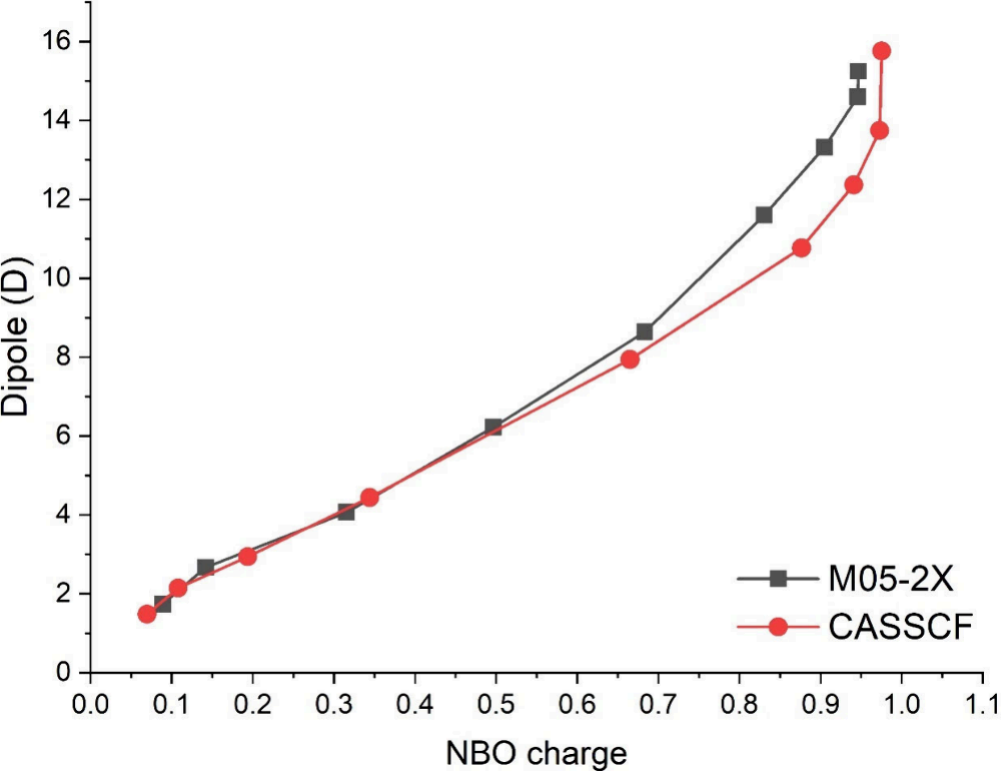
Dipole moment (in D) versus the NBO charge of the cation, obtained at the M05-2X and CASSCF levels with the 6-31+G*(C)/sto-3G(H) and cc-pVDZ(C)/sto-3G(H) basis sets, respectively. From left to right, the C–C distance increases, due to the relaxed scan explained before.

The obtained value for the barrier computed including the CP correction should be taken with caution; as for TS, the NBO charges of the fragments differ significantly from the integer values +1 and −1 (see Figure 4). Such a situation is different from what happens for systems studied previously, in which the NBO charges of the fragments are very close to the integer values mentioned, for both the ion pair and TS.^13,14^

The values of the reaction barrier computed in this work are significantly larger than that predicted for the hydrogen-bonded contact ion pair (HBCIP) formed between chloride and the tropylium cation fused with benzene rings.^13,14^ Besides, the energy difference between the HBCIP and its covalent form^13,14^ is significantly larger than the values between 16.58 and 25.42 kcal/mol obtained in this work. Thus, the two features that are very important to increase the reaction barrier and to decrease the energy difference between the covalent and ionic forms in those systems,^13,14^ that is, the stability of the carbocation^14^ and the doubly ionic hydrogen bond,^28^ are not as effective as the characteristics of the ions shown in Figure 1: their stability and the large *steric hindrance*(4) between them. These two properties seem very promising to yield large barriers for the ion pair → covalent reaction and small energy differences between the ion pair and the covalent form. Thus, one could think of using the following strategy to form a kinetically very stable hydrocarbon ion pair which is, at the same, more stable than its covalent form: choose very stable hydrocarbon ions and systematically increase the steric hindrance between them. It is also possible that the steric hindrance associated with formation of the covalent bond becomes so large that its formation is prevented. Such a strategy has been exploited in the preparation of an ionic solid formed between a substituted fulleride anion and Agranat’s cation.^5,29^ In this context, our results suggest that the mentioned properties of the hydrocarbon ions can also be used to design kinetically and thermodynamically stable hydrocarbon ion pairs *in the gas phase*.

Figure 4 shows the dipole moments of the system versus the NBO charges (of the cation), computed at both the M05-2X and CASSCF levels for the structures obtained from the relaxed scan performed along the C–C coordinate explained in Figures 1 and 2. It is clear from this plot that the NBO charge increases as the polarity of the system increases. Besides, both quantities increase as the C–C distance increases, along the relaxed scan.

### Characterization of the Ion Pair in the Gas Phase

All structures have been obtained at the M05-2X level. The value of the RMSD parameter, obtained through comparison between the isolated (optimized) cation and the cationic fragment as it is in the ion pair is 0.296 Å. On the other hand, if the same fragment is compared to its optimized radical (that is, the reduced form), a value of 0.525 Å is obtained. In the case of the anionic fragment (the simplified Kuhn’s anion, see Figure 1) as it is in the ion pair the corresponding values are 0.279 and 0.286 Å, respectively. Therefore, the structures of the fragments in the ion pair resemble more the structures of the isolated ions than those of the isolated radicals, that is, the reduced and oxidized forms for the cation and anion, respectively. However, it is important to point out that the structural effect due to the reduction of the cation is much more pronounced than the structural effect associated with oxidation of the anion, as can be seen through the comparison between the differences of 0.229 (0.525–0.296) and 0.007 Å (0.286–0.279).

The NBO charges of the cations of the ion pair are 0.946 and 0.975 at the M05-2X and CASSCF levels, respectively (see Figure 4), which are close to the “ideal” value of 1.000. The dipole moment of the ion pair is very large, with a value of 15.250 and 15.762 D, again at the M05-2X and CASSCF levels, respectively. These values are significantly larger than those of other gaseous ion pairs studied previously^13,14,30−34^ and much larger than that of the covalent form, 1.734 and 1.484 D, at the same two levels.

At the CASSCF level the electronic configuration obtained for S_0_ can be described as a combination of the closed shell configuration (with a weight of 92.7%) and the configuration generated by the single HOMO → LUMO excitation (with a weight of only 2.7%). The remaining configurations contribute with weights smaller than 1%.

The diradical character can be estimated by the occupation number of the lowest unoccupied natural orbital (LUNO).^35,36^ Its occupation number, obtained at the CASSCF level with the cc-pVDZ(C)/sto-3G(H) basis set, leads to a diradical character of only 7.8%. This low value is consistent with the large weight of 92.7% of the closed shell configuration. For the structures of the relaxed scan, the maximum value of the diradical character is only 10.4%. Figure S4 shows the occupation numbers of the natural orbitals versus the C–C distance, for the structures of the relaxed scan explained previously. The large singlet–triplet gap of 1.84 and 2.41 eV (at the M05-2X and CASSCF levels, respectively) are consistent with the small diradical character of the ion pair. The optimized orbitals obtained for T_1_ at the CASSCF level are shown in Figure S2 in the SI. The optimization of the ion pair at the UDFT level yielded essentially the same structure and energy as those obtained at the restricted DFT level. This outcome is also consistent with the small diradical character of the ion pair.

The computed energy differences between S_0_ and S_1_ are 2.32 (∼533 nm) and 2.84 eV (∼437 nm) at the TD-DFT (with the M05-2X functional) and CASSCF levels, respectively. At the TD-DFT level S_1_ is mainly composed of the singly excited HOMO → LUMO and HOMO → LUMO+2 configurations, with weights of 40.5 and 7.8%, respectively. The Kohn–Sham orbitals HOMO and LUMO are more localized in the anionic and cationic fragments, respectively, but the LUMO orbital has a significant contribution from the anionic fragment. Thus, according to the TD-DFT results S_1_ has some charge recombination character,^13,14^ which differs from the CASSCF result for S_1_, discussed below.

At the CASSCF level, S_1_ is mainly composed of the closed shell configuration and a doubly excited configuration, HOMO–1 → LUMO, with weights of 46.6 and 42.3%, respectively. Although the active orbitals have been optimized for S_1_, they remain localized in the anionic fragment, as those of S_0_. The optimized orbitals obtained for S_1_ are shown in Figure S3 of SI. However, they are not adequate for S_0_, as they lead to a very different electronic configuration, as compared to that obtained from the orbitals optimized for S_0_. As discussed before the latter set yields a combination formed by the closed shell configuration (which is highly dominating) and that generated by the single HOMO → LUMO excitation. On the other hand, if one uses the set optimized for S_1_ one obtains a highly multiconfigurational ground state, containing eight configurations with weights larger than 1%. The three largest weights correspond to the configuration generated by the single HOMO–1 → LUMO excitation (∼46%), the closed shell configuration (∼21%), and that generated by the double HOMO–1 → LUMO excitation (∼21%). Besides, the energy of S_0_ is smaller if it is computed with the set of orbitals optimized for this state, as expected from the variational theorem. Therefore, the energy difference of 2.84 eV has been computed using the lowest value for the energy of S_0_.

The experimental value obtained for the lowest energy absorption band in DMSO is 1.78 eV (696 nm).^3^ For the DMSO solution of the salt formed by the Kuhns’ anion and the cation shown in Figure 1, all four UV–vis absorption bands are due to the anion.^3^

The same holds for the S_1_ state of the system studied in this work at the CASSCF level, although the computed value is 1.06 eV larger. At the TD-DFT level, the value is 0.54 eV larger, but at this level the S_1_ state has some charge recombination character, as explained before. Although one does not expect an agreement between the experimental and theoretical values, mainly due to the solvent effect and to the influence of the cation, it is important to point out that even for the gaseous ion pair the lowest energy band is, according to the highest level (CASSCF) result, mainly due to the anion, despite the strong influence of the cation.

**Figure 5:**
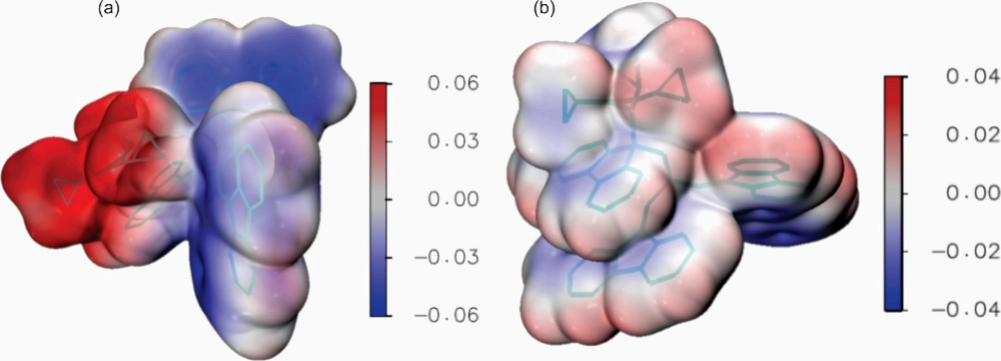
MEP for the ion pair (a) and covalent structure (b), computed at the M05-2X level with the 6-31+G*(C)/sto-3G(H) basis set. The plots shown have been generated by the Multiwfn^37^ and VMD softwares.^38^ The values are given in atomic units.

**Figure 6:**
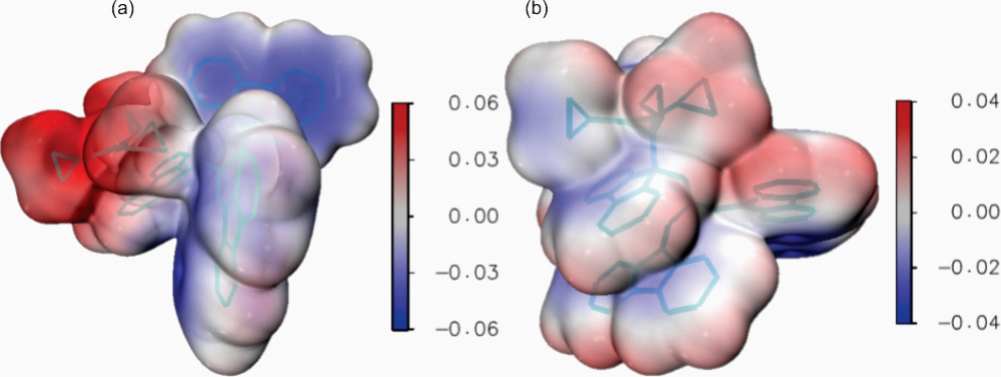
MEP for the ion pair (a) and covalent structure (b), computed at the CASSCF level with the cc-pVDZ(C)/sto-3G(H) basis set. The plots shown have been generated by the Multiwfn^37^ and VMD softwares.^38^ The values are given in atomic units.

Figures 5 and 6 show the molecular electrostatic potential (MEP) computed at the M05-2X and CASSCF levels, respectively, for the ion pair and the covalent structure. Both plots show that at both levels one has: (i) a significantly wider range of values for the ion pair; (ii) larger (and positive) values for the cationic region of the ion pair than for the cationic region of the covalent structure; (iii) smaller (and negative) values for the anionic region of the ion pair than for the anionic region of the covalent structure. Therefore, data in Figures 5 and 6 are consistent with the larger dipole moment of the ion pair and with its larger charge separation, in comparison with the corresponding values for the covalent form (see also Figure 4).

As mentioned previously, an EDA calculation^24^ has been performed to characterize the interaction between the ions, in the ion-pair structure. The total interaction energy at the DFT level and its contributions are given in Table 1. The most important attractive contribution is due to the electrostatic term, as expected. The dispersion energy is the second most important attractive term, and it corresponds to ∼62% of the electrostatic term. To estimate the influence of the cyclopropyl substituents (see Figure 1) on the dispersion energy the EDA calculation has been repeated after replacing these groups by hydrogen atoms and keeping the rest of the structure frozen. With this alteration the dispersion energy changes to −13.66 kcal/mol, now corresponding to only ∼21% of the electrostatic term, which clearly shows the importance of the cyclopropyl substituents. Besides, only the covalent form has been obtained after replacing the cyclopropyl substituents by hydrogen atoms and performing a full geometry optimization. Thus, the steric hindrance due to the cyclopropyl substituents is essential for the existence of the ion pair.

**Table 1:** Interaction Energy and Its Contributions, as Obtained from an EDA Calculation, between the Ions Shown in Figure 1 (in the Ion Pair Structure)^a^

Energy term	Value in kcal/mol
Total interaction energy	–74.83
Electrostatic energy	–68.34
Exchange energy	–15.37
Repulsion energy	68.79
Polarization energy	–13.28
DFT dispersion energy	–46.64

a The values have been obtained at the DFT level with the mixed 6-31+G*(C)/sto-3G(H) basis set.

### Solvent Effects

**Figure 7:**
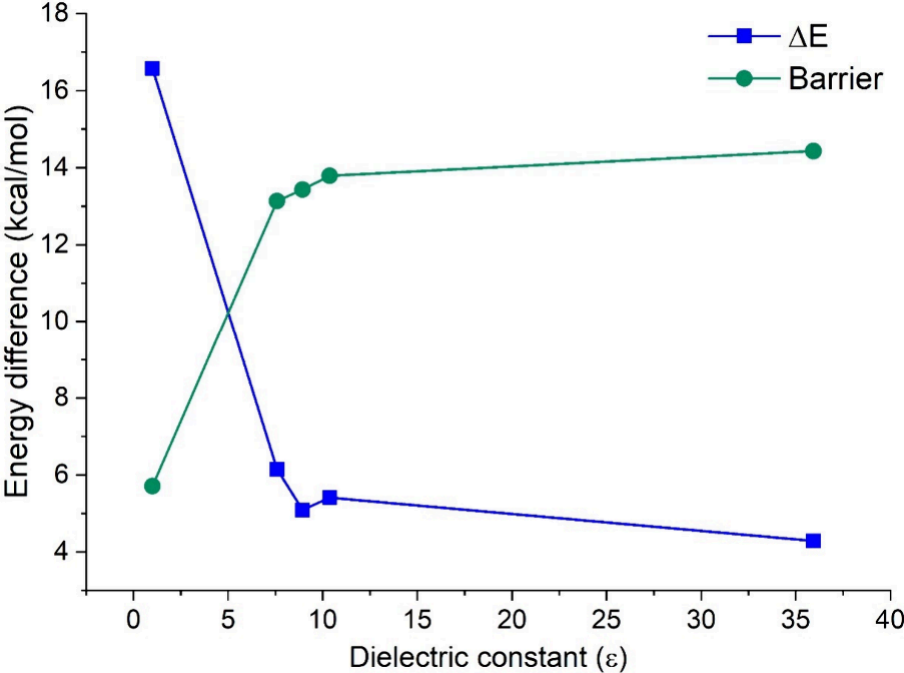
Δ*E* and the barrier, from the ion pair to the covalent form, computed at the M05-2X level (with the 6-31+G*(C)/sto-3G(H) basis set) in several nonprotic solvents of increasing dielectric constants.

As explained before the solvent effects have been considered only at the DFT level. Figure 7 shows the barrier for the ion pair → covalent reaction and the values of Δ*E*, computed in gas phase and in the four solvents studied in this work. It is clear from Figure 7 that the studied solvents impart a significant influence in Δ*E*, decreasing it from 16.58 to 4.29 kcal/mol, from the gas phase to acetonitrile. The general trend is a decrease of Δ*E* as the dielectric constant of the solvent increases, but from dichloromethane to EDC Δ*E* slightly increases, from 5.09 to 5.41 kcal/mol. However, this very small difference can be a limitation of the CPCM model to differentiate specific solute–solvent interactions in the case of these two solvents. Further investigations are required to test this hypothesis. The very large differences of ∼13.5 and 14.3 D (at the M05-2X and CASSCF levels, respectively) between the dipole moments of the ion pair and the covalent form (see Figure 4) explains the much larger stabilization of the ion pair in the solvents of larger polarity, and thus the significant decrease in the Δ*E* values.

The dipole moment of the TS is between those of the covalent form and the ion pair (see Figure 4) but is still much smaller than that of the ion pair, with differences of ∼6.6 and 7.8 D, at the M05-2X and CASSCF levels, respectively. Such differences explain the increase of the studied barrier as the dielectric constant of the solvent increases (see Figure 4), reaching the maximum value of ∼14.4 kcal/mol. Therefore, increasing this latter property of the studied solvents one can increase the kinetic and thermodynamic stability of the ion pair. However, even acetonitrile (with a dielectric constant of 35.94) was not enough to yield an ion pair which is more stable than the covalent form, although Δ*E* has been reduced to only 4.29 kcal/mol. In this context it is interesting to investigate nonprotic solvents with larger values of the dielectric constant. This work is in progress in our group. The Cartesian coordinates of the three structures optimized in all four solvents are given in Tables S2–S5 in the SI.

### Possible Formation of the Ion Pair from the Radicals, in HNDs

As discussed previously, even for kinetically less stable ion pairs,^13,14^ the HND technique is potentially adequate not only to induce the formation of the ion pair studied in this work but also for its trapping and detection. Thus, only a brief discussion will be given here.

The radicals that can form the ion pair studied in this work are the reduced and oxidized forms of the ions shown in Figure 1(a) and 1(b), respectively (named R_1_· and R_2_·). The ion pair can then be formed from these radicals via the harpoon mechanism.^13^ At the M05-2X level the difference between the ionization potential of R_1_· and the electron affinity of R_2_· is 2.63 eV, which yields a critical distance of ∼5.47 Å for the electron transfer between these radicals.^13^ As this value is larger than the distance between the centers of masses of the ions (5.085 Å) in the ion pair, the charge-transfer reaction can take place before formation of the ion pair, which is consistent with the harpoon mechanism (step (b) of the mechanism discussed later).

Assuming that the radicals can be generated inside the HNDs through proper pyrolysis sources,^39,40^ the very low pressures in the pyrolysis region minimize recombination of the newly formed radicals and are optimized for the pickup of single molecules (or radicals).^39−42^ After the pickup, the molecular degrees of freedom of the hot radicals are rapidly cooled (<1 ns) to the droplet temperature (0.4 K) via evaporation of He atoms,^43,44^ which is essential for the trapping. The two characteristics of the HNDs, (i) very fast cooling and (ii) sequential capture of the reactive species R_1_· and R_2_· , can generate a pair of reacting radicals with minimum energy, minimizing the possibility of side reactions, as isomerizations and hydrogen shifts. Once one has a reactive encounter between the different radicals, induced by the sequential pickup, the ion pair (R_1_^+^R_2_^–^) or the covalent structure (R_1_–R_2_) can be formed. The former species can be probed within the HNDs through determination of its very large dipole moment via the Stark effect.^45−50^ As the formation of either the ion pair or the covalent form are energetically favorable, starting from the radicals, their formation within the HNDs is expected to cause evaporation of He atoms.^43,44^ The formation of the covalent form is 25.42 kcal/mol more favorable (at the CASSCF level) than the formation of the ion pair. Thus, it should lead to the evaporation of ∼1816 extra He atoms,^43,44^ as compared to the number resulting from the formation of the ion pair. Therefore, mass measurements allow one to distinguish between occurrence of the R_1_· + R_2_· → R_1_^+^R_2_^–^ or the R_1_· + R_2_· → R_1_–R_2_ reaction after a reactive encounter between the radicals.^51^

The following kinetic model, published recently,^52^ is consistent with transient formation of the *R*_1_^+^*R*_2_^–^ species from R_1_· and R_2_·:
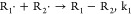
a
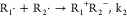
b
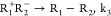
c

As the products of the elementary steps above are much more stable than the reactants, these three reactions are considered irreversible.^52^ Additionally, the reactions R_1_· + R_1_· → R_1_–R_1_ and R_2_· + R_2_· → R_2_–R_2_ can be neglected due to the very low pressures in the pyrolysis region, as explained above. It can be shown that, for a given value of [R_1_·]_0_ (the initial concentration of R_1_·, assuming [R_1_·]_0_ = [R_2_·]_0_), the larger is the value of k_2_, the larger is the maximum concentration of the ion-pair ([R_1_^+^R_2_^-^]_max_) and the slower is its decay.^52^ Therefore, if k_2_ is large enough, the trapping and detection (inside HNDs) of the kinetically metastable ion-pair studied in this work seems to be possible, due to the unique characteristics of the HND technique explained before. However, only accurate calculations of the three rate constants (which unfortunately are prohibitive, due to the size of the radicals) can lead to more reliable conclusions concerning the value of [R_1_^+^R_2_^-^]_max_ and the time elapsed until this value is reached (*t*_max_). With this information in hand, one can better decide whether [R_1_^+^R_2_^-^]_max_ and t_max_ are consistent with the trapping and detection inside HNDs. For instance, a rough estimate yields [R_1_^+^R_2_^-^]_*max*_ ∼ 0.19[R_1_·]_0_ and t_max_ = 1.20 ms.^52^ A reaction forming a transient ion pair via the harpoon mechanism has already been suggested to take place in HNDs.^53^

## Conclusions

The ion pair formed between the tricyclopropylcyclopropenylium cation and a simplified Kuhn’s anion (see Figure 1) has been studied at the DFT and CASSCF levels, either in the gas phase or in four nonprotic solvents of increasing dielectric constants. The solvent effect has been modeled through the CPCM continuum solvation model. This is the first example of a realistic hydrocarbon ion pair studied through quantum chemistry methods with expected good accuracy. An energy decomposition analysis indicates that the most important attractive contribution associated with formation of the ion pair is due to the electrostatic term. The dispersion energy is the second most important attractive term, comprising ∼62% of the electrostatic term, and this term is mainly due to the three cyclopropyl substituents. The barrier from the ion pair to the covalent structure varies from ∼4.8 to 14.4 kcal/mol, while the energy difference between the ion pair and the covalent form varies from ∼4.3 to 25.4 kcal/mol. The general trend is a decrease of this latter energy difference and an increase of the mentioned barrier, as the dielectric constant of the solvent increases. The smallest energy difference and the largest barrier have been obtained in acetonitrile, the studied solvent having the largest dielectric constant. The ion pair is characterized by its dipole moment, electrostatic potential, NBO charges, structure, occupation numbers of the natural orbitals, vertical excitation energy to S_1,_, and singlet–triplet gap. Its possible formation (from the corresponding radicals), trapping, and subsequent identification, in HNDs, can be possible, depending on the rate constant associated with its formation. Besides, the formation of a transient ion pair via the same (harpoon) mechanism suggested in this work has already been suggested to take place in HNDs.^53^ This latter outcome along with a kinetic model published recently^52^ can shed more light on the viability of formation (from the radicals), followed by the trapping and detection of the studied hydrocarbon ion pair in HNDs.

The results obtained in this work suggest that if one uses very stable hydrocarbon ions with a very large steric hindrance between the opposite ions, it is possible to obtain kinetically and thermodynamically stable hydrocarbon ion pairs *in the gas phase*.

## Supporting Information Available

Optimized CAS(6,6) orbitals along the relaxed scan, and for the triplet and S_1_ states; Cartesian coordinates (in Å) of the optimized structures of the ion pair, TS and the covalent form, in gas phase and in the four solvents studied in this work; occupation numbers of the natural orbitals (at the CASSCF level) versus the C–C distance, for the structures of the relaxed scan (PDF)
