# Supplementary material for: Theoretical Study of an Authentic Hydrocarbon Ion Pair
Source: ACS Omega. 2024 Aug 2;9(32):34981–9. doi: 10.1021/acsomega.4c04914 (PMC11325399; doi:10.1021/acsomega.4c04914)
Supplement: Supplementary file 1 — ao4c04914_si_001.pdf [file ao4c04914_si_001.pdf]

Theoretical study of an authentic hydrocarbon ion pair

Elizete Ventura[a], Gessenildo Pereira Rodrigues[a], Ezequiel Fragoso Vieira Leitão[b] and Silmar Andrade do Monte[a*]

[a]Departamento de Química, CCEN, Universidade Federal da Paraíba, 58059-900, João Pessoa, Brazil
[b]Unidade Acadêmica de Ciências Exatas e da Natureza, Universidade Federal de Campina Grande, Cajazeiras, PB, Brazil

Index Page

Index	Page
Figure S1. Optimized orbitals at the CAS(6,6) level along the relaxed scan.	2
Figure S2. Optimized orbitals at the CAS(6,6) level for the triplet state.	3
Figure S3. Optimized orbitals at the CAS(6,6) level for S_1.	3
Table S1. Cartesian coordinates (in Å) of the optimized structures of the ion pair, TS and the covalent form.	4
Table S2. Cartesian coordinates (in Å) of the optimized structures of the ion pair, TS and the covalent form in THF.	10
Table S3. Cartesian coordinates (in Å) of the optimized structures of the ion pair, TS and the covalent form in dichloromethane.	13
Table S4. Cartesian coordinates (in Å) of the optimized structures of the ion pair, TS and the covalent form in EDC.	16
Table S5. Cartesian coordinates (in Å) of the optimized structures of the ion pair, TS and the covalent form in acetonitrile.	19
Figure S4. Occupation numbers of the natural orbitals versus the C–C distance, for the structures of the relaxed scan.	22

Figure S1. Optimized orbitals at the CAS(6,6) level along the relaxed scan. The mixed cc-PVDZ(C)/sto-3G(H) basis set has been used. The covalent, ts and ion pair structures are also indicated. The hydrogens have been omitted for clarity.

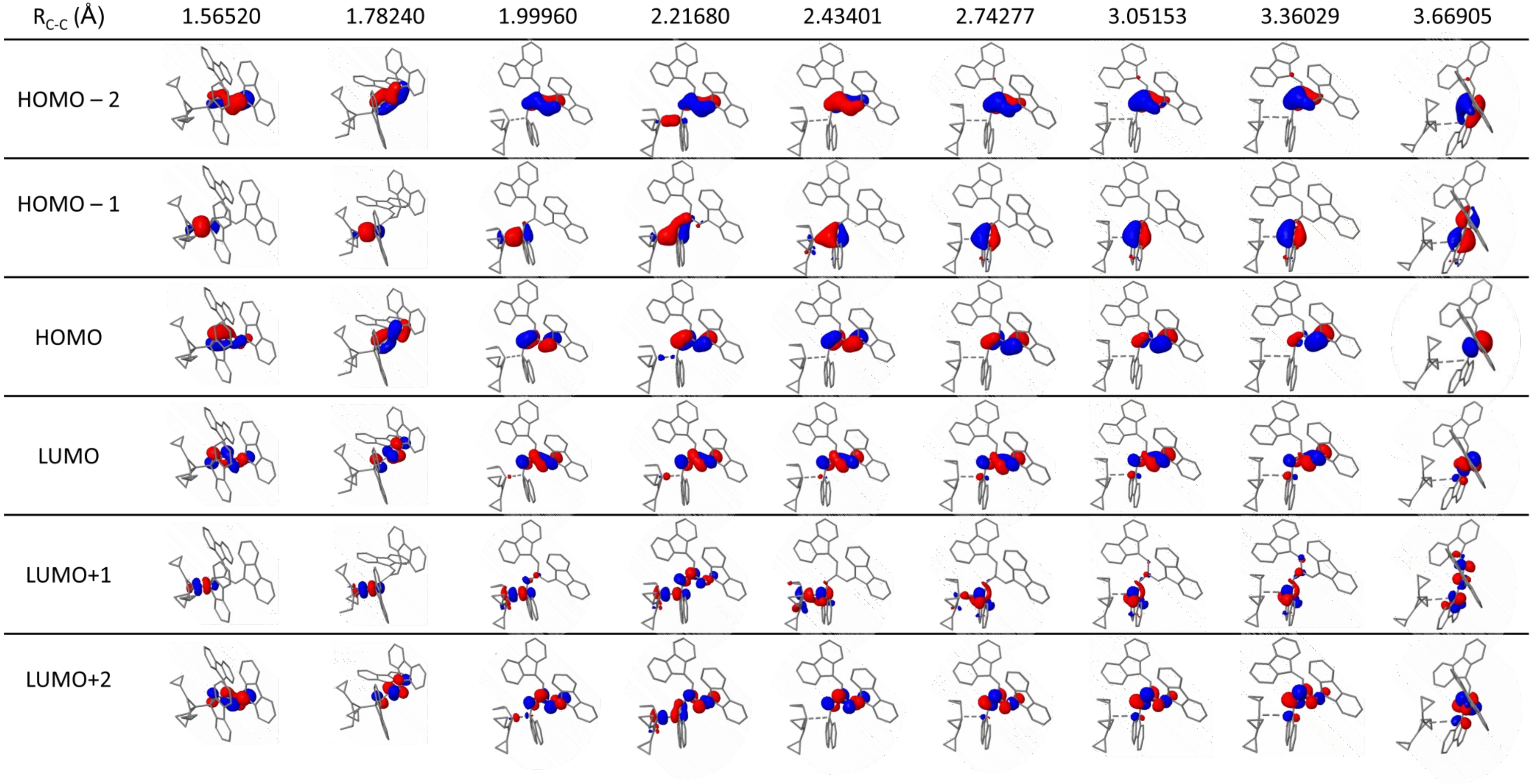

Figure S2. Optimized orbitals at the CAS(6,6) level for the triplet state. The mixed cc-PVDZ(C)/sto-3G(H) basis set has been used.

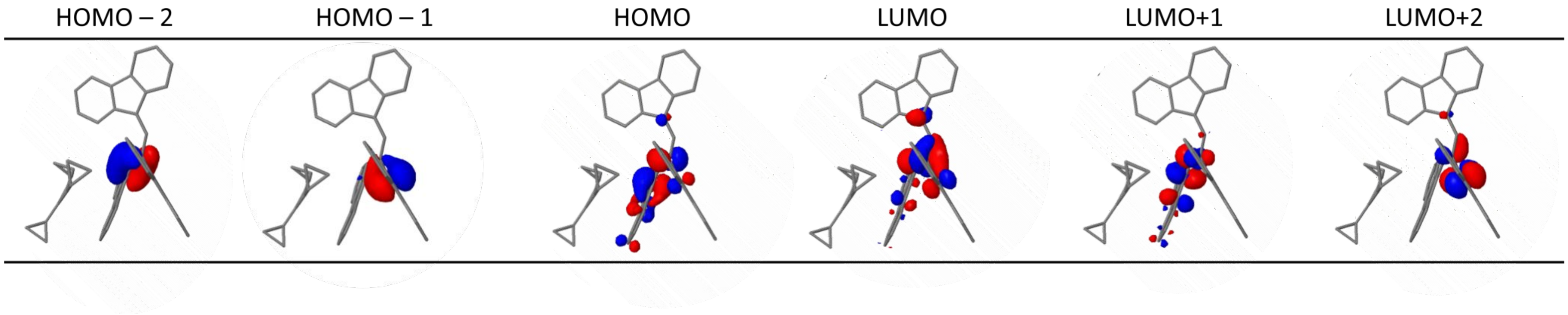

Figure S3. Optimized orbitals at the CAS(6,6) level for S_1. The mixed cc-PVDZ(C)/sto-3G(H) basis set has been used.

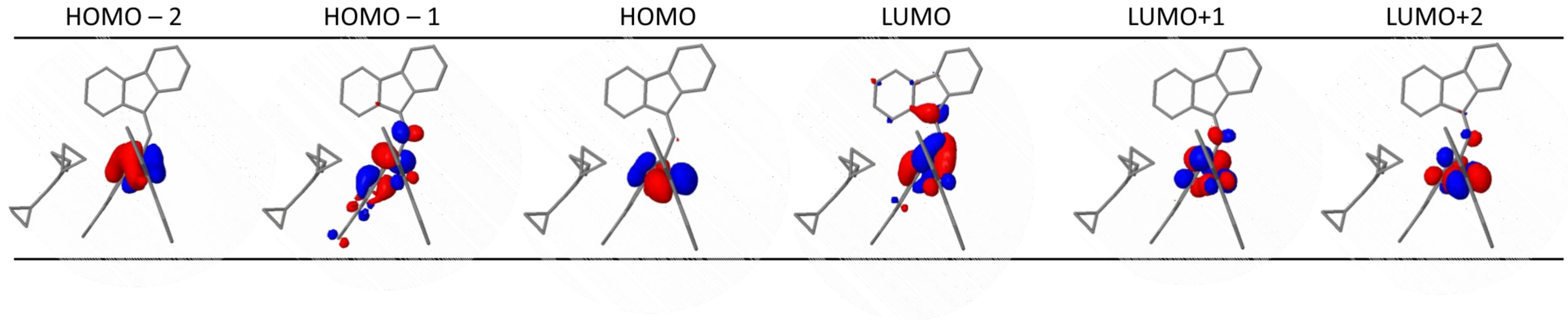

Table S1. Cartesian coordinates (in Å) of the optimized structures of the ion pair, ts and the covalent form. The calculations have been performed at the M05-2X level with the 6-31+G*(C)/sto-3G(H) basis set.

ion pair				ts				covalent			
C	1.33088800	-0.42036900	-1.71758700	C	1.49720600	0.36331100	-0.82402400	C	-1.08152500	-1.14331500	0.58449700
C	3.12583400	-0.78881900	1.46114400	C	2.89303900	-1.23121100	0.37327800	C	-1.36955600	-2.68117900	0.54129700
C	-0.97528000	-4.87705400	1.96472500	C	-1.16691100	-4.30736800	2.59557300	C	4.87105600	-1.68512600	-2.66038500
C	-0.15314200	-3.79580400	1.62807800	C	-0.66473000	-3.00369700	2.53416200	C	3.48579100	-1.57223900	-2.53116400
C	-0.61055400	-2.77908500	0.79155600	C	-0.95838900	-2.17434800	1.45123600	C	2.91606400	-1.00637000	-1.39008000
C	-1.91166600	-2.82899700	0.28297200	C	-1.76319800	-2.65704600	0.41827800	C	3.74376600	-0.53106300	-0.37519200
C	-2.74527900	-3.91337900	0.65606800	C	-2.31820600	-3.95275200	0.51976700	C	5.14451000	-0.63350000	-0.52313100
C	-2.27910600	-4.93504100	1.47913300	C	-2.00701800	-4.78498800	1.59085600	C	5.71034800	-1.21386400	-1.65184000
H	-0.59862200	-5.66565500	2.60869400	H	-0.92177800	-4.94027800	3.44286900	H	5.29570600	-2.13648300	-3.55152900
H	0.85934500	-3.75241200	2.02287300	H	-0.04167800	-2.63055000	3.34237500	H	2.83942800	-1.93169400	-3.32599100
H	0.03965900	-1.95181900	0.53378200	H	-0.56456500	-1.16348300	1.40983900	H	1.83922000	-0.92745300	-1.30975500
H	-2.92979000	-5.76074600	1.75193000	H	-2.43302900	-5.78156200	1.65896400	H	6.78875000	-1.28815000	-1.75366000
C	-2.70122600	-1.88244900	-0.52543700	C	-2.33244700	-1.99024300	-0.77515200	C	3.45529200	0.18409000	0.89367400
C	-4.04951200	-2.46810400	-0.60747800	C	-3.29825700	-2.95361000	-1.33978700	C	4.78486300	0.48229700	1.48487100
C	-5.20452400	-2.00891800	-1.24268800	C	-4.13692100	-2.85199700	-2.44664900	C	5.12449700	1.12270500	2.67224600
C	-6.36642100	-2.77215800	-1.16676500	C	-4.93968500	-3.93984200	-2.78502900	C	6.47254200	1.26282700	3.00112500
C	-6.38424500	-3.99195100	-0.47768000	C	-4.89624100	-5.12325100	-2.03920700	C	7.47067100	0.76424200	2.15874600
C	-5.23447900	-4.46125500	0.15179700	C	-4.05247000	-5.23378400	-0.93562200	C	7.13509200	0.11736100	0.97024900
C	-4.07275500	-3.69581600	0.08476700	C	-3.25813500	-4.14480700	-0.59015300	C	5.79062300	-0.01912100	0.64240100
H	-5.20563100	-1.07390100	-1.79462700	H	-4.17107200	-1.94351400	-3.04005000	H	4.36288900	1.51037800	3.34163700
H	-7.27026400	-2.41962200	-1.65433800	H	-5.60463300	-3.86977300	-3.64020000	H	6.74937300	1.76345900	3.92359100
H	-7.29955100	-4.57416200	-0.43746300	H	-5.52556400	-5.96085900	-2.32316000	H	8.51423500	0.88185800	2.43322300
H	-5.24695200	-5.40780200	0.68447000	H	-4.02023900	-6.15254900	-0.35762300	H	7.91053600	-0.27103600	0.31707900
C	-4.53932500	3.00061500	2.46088500	C	-5.69561100	1.66426400	2.07990700	C	0.62202400	3.35162600	-3.61734400
C	-4.08961100	1.69253700	2.25417800	C	-4.84659200	0.56792800	1.91299800	C	1.40821000	2.38110500	-2.99232400
C	-3.23050600	1.38379200	1.20042500	C	-3.70226700	0.66098200	1.11999300	C	1.18559200	2.03432100	-1.65913600
C	-2.80972300	2.39834500	0.33367200	C	-3.41661100	1.86352100	0.47527900	C	0.16105100	2.66157700	-0.96201400
C	-3.25111500	3.72382700	0.56890100	C	-4.25474700	2.98072000	0.68768300	C	-0.60764700	3.66447300	-1.58430800
C	-4.11514100	4.02488100	1.61766200	C	-5.39582800	2.88468600	1.47502400	C	-0.38834300	4.00845000	-2.91234300
H	-5.21738800	3.21719200	3.28061400	H	-6.58342800	1.57092700	2.69788700	H	0.80907900	3.60921500	-4.65550400
H	-4.42098100	0.89715600	2.91516200	H	-5.07347500	-0.37153000	2.40738200	H	2.20708400	1.89235800	-3.54246700

H	-2.90550900	0.35854400	1.06787500	H	-3.05147600	-0.19829300	1.02272400	H	1.81592300	1.29794400	-1.17924800
H	-4.44795100	5.04591200	1.78068800	H	-6.03635600	3.74803400	1.62918100	H	-0.98516500	4.77757800	-3.39401500
C	-1.86809400	2.41358400	-0.80640900	C	-2.27911700	2.28208100	-0.37517400	C	-0.31334000	2.49046900	0.42914600
C	-1.77837800	3.82571900	-1.22411100	C	-2.46620500	3.73442400	-0.59624900	C	-1.36890700	3.51537600	0.61450300
C	-1.03485000	4.42903000	-2.23867800	C	-1.68428300	4.65288100	-1.29055900	C	-2.15541000	3.81068400	1.72047600
C	-1.13447100	5.80630700	-2.42277600	C	-2.11160500	5.97811200	-1.36853400	C	-3.13454700	4.79908300	1.60477700
C	-1.97351400	6.58133600	-1.61237300	C	-3.30896000	6.38073400	-0.76849200	C	-3.32793300	5.47381700	0.39625000
C	-2.72205400	5.98533600	-0.59974800	C	-4.09634000	5.46385700	-0.07283500	C	-2.54330000	5.17471100	-0.71883600
C	-2.61843800	4.61003100	-0.40911000	C	-3.66673700	4.14373700	0.01157200	C	-1.56436700	4.19508800	-0.60182900
H	-0.39006800	3.84188800	-2.88606700	H	-0.75409500	4.35343400	-1.76408800	H	-2.02523800	3.27956200	2.65864600
H	-0.55902300	6.28351900	-3.21022400	H	-1.50762100	6.70501000	-1.90260800	H	-3.75372100	5.04390400	2.46241400
H	-2.04351800	7.65188500	-1.77851100	H	-3.62714500	7.41588300	-0.84507000	H	-4.09540300	6.23845000	0.32468000
H	-3.37480500	6.58573600	0.02729200	H	-5.02524800	5.77841500	0.39358000	H	-2.69892800	5.69778300	-1.65776100
C	-1.09807500	1.43828400	-1.36956800	C	-1.22831600	1.59139400	-0.87171800	C	-0.00402900	1.54892800	1.33438100
H	-0.33980300	1.78373600	-2.07278900	H	-0.43961100	2.17906600	-1.33909400	H	-0.49581500	1.59965200	2.30555700
C	4.42724400	-2.60420100	-3.63900400	C	4.84273300	1.02988100	-3.33896300	C	-3.92244100	0.72295300	3.21869400
C	3.14740900	-3.18546800	-3.63423200	C	3.95248000	0.00981300	-3.72646900	C	-2.92620400	-0.05702300	3.81186100
C	2.06683900	-2.54502800	-3.03826300	C	2.82285900	-0.28176500	-2.97367700	C	-1.95603300	-0.68941400	3.02998200
C	2.26528000	-1.30760300	-2.40867500	C	2.58101400	0.43919800	-1.79367600	C	-1.99560600	-0.53297100	1.65065000
C	3.56251300	-0.74306500	-2.39405900	C	3.48021200	1.46837200	-1.40805000	C	-2.97561300	0.27913000	1.06053700
C	4.63701900	-1.37731200	-3.01998200	C	4.60142200	1.76705400	-2.18741900	C	-3.94730300	0.90494000	1.83617000
H	5.24884000	-3.11248100	-4.13397700	H	5.70470000	1.25573400	-3.95936300	H	-4.67011200	1.20662300	3.84021300
H	2.99470900	-4.14300200	-4.12327900	H	4.13917200	-0.53859600	-4.64535200	H	-2.90501200	-0.17371500	4.89123400
H	1.08275100	-3.00273700	-3.07711500	H	2.12365900	-1.04318700	-3.30828200	H	-1.17585700	-1.27946600	3.49919600
H	5.62470100	-0.92296400	-3.02242600	H	5.27217800	2.57228500	-1.89851000	H	-4.69619800	1.54409300	1.37736600
C	2.13828000	0.70636800	-1.24112700	C	1.71651000	1.46417200	0.10383100	C	-1.60565900	-0.45600500	-0.67288700
C	1.85320400	1.84149000	-0.46039700	C	0.98778800	1.90273000	1.22207500	C	-1.12588200	-0.54096300	-1.97194600
C	2.87740900	2.70562900	-0.08602500	C	1.48905900	2.93616600	1.99970400	C	-1.74646800	0.20930400	-2.97325400
C	4.20639200	2.47573200	-0.47375800	C	2.72280900	3.54147600	1.69840000	C	-2.83452200	1.03278800	-2.67481800
C	4.51362500	1.36306300	-1.25019800	C	3.45955600	3.11803600	0.60027500	C	-3.32761700	1.11020800	-1.37280400
C	3.49397300	0.49003500	-1.63161900	C	2.95598000	2.08657000	-0.20019500	C	-2.71093900	0.35459800	-0.38015200
H	0.84080400	2.07279200	-0.15393900	H	0.02962700	1.45442700	1.46915900	H	-0.27471100	-1.17167100	-2.21215700
H	2.62993700	3.59007900	0.49591200	H	0.91357400	3.28952300	2.85066100	H	-1.36266600	0.17202400	-3.98812900
H	4.98695900	3.17463300	-0.18734600	H	3.08969800	4.35315300	2.31916500	H	-3.28862500	1.62922500	-3.46019700
H	5.53709200	1.18020500	-1.56877600	H	4.40786000	3.59105300	0.35728900	H	-4.16028800	1.76657700	-1.13528600
C	-0.01087700	-0.75959300	-1.56932900	C	0.26816700	-0.34608400	-1.05794200	C	0.39092500	-0.83681200	0.81692500

H -0.24331600 -1.79810300 -1.80759100	H 0.35139200 -1.36541000 -1.43640400	H 1.08740400 -1.67104700 0.74376400
C -2.40413500 -0.64184600 -1.01756800	C -2.13009700 -0.73619000 -1.24014900	C 2.30728000 0.60384100 1.46497600
H -3.26058300 -0.04466500 -1.33424100	H -2.84379800 -0.34524400 -1.96632400	H 2.42536700 1.24396800 2.33959100
C -1.13646800 0.00796700 -1.21320200	C -1.00766400 0.14903200 -0.93258900	C 0.89307300 0.37496000 1.12264700
C 3.89517300 0.17572600 2.05929900	C 4.19985400 -0.72474400 0.34558000	C -2.63094700 -3.11811000 -0.13590000
C 2.52711600 0.23836000 2.16432800	C 3.44175700 -0.51087400 1.44167000	C -1.53004500 -3.42252300 -0.76064700
C 1.37023100 0.85098600 2.75488400	C 3.32360500 -0.10072200 2.82680200	C -0.82876800 -4.06466900 -1.86991600
C 0.01141800 0.72252000 2.07734400	C 1.97580400 -0.17733000 3.49184100	C 0.65320200 -3.84662900 -2.04707400
C 0.26875200 -0.08097000 3.28588300	C 2.99033400 -1.21651300 3.81844400	C 0.16196200 -5.16778800 -1.54915200
H 1.56529500 1.76148900 3.30994200	H 4.01104700 0.67229400 3.15245500	H -1.41728500 -4.19920100 -2.77363200
H -0.01685200 0.22352400 1.11286300	H 1.14359000 -0.45417600 2.85296800	H 1.14654500 -3.20039900 -1.32767500
H -0.62351200 1.60047800 2.15057500	H 1.75507800 0.59378700 4.22237400	H 1.03733000 -3.78360500 -3.06038300
H -0.17430700 0.21972000 4.22989700	H 3.50000100 -1.17768900 4.77585900	H 0.18843700 -6.02124800 -2.21920800
H 0.42640000 -1.14952900 3.16691500	H 2.85604900 -2.21360400 3.40727900	H 0.31481600 -5.39853900 -0.49943400
C 3.07581800 -2.05067100 0.77137200	C 2.18647200 -2.47292800 0.05766600	C -0.88750500 -3.42147400 1.77947700
C 4.32801300 -2.54262200 0.07791700	C 2.53742200 -3.23439600 -1.19309800	C -1.87104900 -3.89638400 2.81676200
C 3.95032300 -3.16221900 1.36948700	C 3.00779900 -3.75010600 0.13039600	C -1.17044300 -4.89056200 1.93535500
H 2.12053300 -2.33450000 0.34951900	H 1.16780300 -2.54194800 0.41668800	H 0.07607400 -3.09528600 2.16694200
H 5.16948400 -1.85600600 0.05086600	H 3.27863400 -2.78878800 -1.84932100	H -2.92104800 -3.70019800 2.61690800
H 4.15748500 -3.09059200 -0.84388200	H 1.72908900 -3.75612100 -1.69678900	H -1.59442200 -3.84369600 3.86614600
H 3.50076900 -4.15030100 1.36832000	H 2.51308300 -4.61440400 0.56257400	H -0.39852500 -5.51455500 2.37598800
H 4.52266900 -2.91788100 2.25964300	H 4.06489300 -3.65655200 0.36503100	H -1.75655200 -5.37065000 1.15587700
C 5.18175100 0.70123000 2.44985300	C 5.51044300 -0.69109300 -0.27689400	C -4.08822000 -3.01245400 -0.10214500
C 5.26561800 2.17355100 2.79794500	C 6.51082300 0.31176600 0.23702700	C -4.76066900 -2.28822900 -1.25189400
C 5.31181800 1.17774900 3.89622200	C 6.68950100 -1.12863800 0.58282300	C -4.90186800 -3.77266800 -1.12141900
H 6.04513300 0.23403100 1.99110300	H 5.53172000 -0.93019000 -1.33501600	H -4.52531600 -2.85908100 0.87961700
H 4.35933000 2.75424100 2.65473200	H 6.16246400 0.99181600 1.00984800	H -4.10275500 -1.89252900 -2.02085100
H 6.18245300 2.67509500 2.50591600	H 7.17094000 0.75414300 -0.50185700	H -5.61244800 -1.66003500 -1.00893900
H 6.26097400 0.96726300 4.37824800	H 7.47015800 -1.69242300 0.08187000	H -5.85711200 -4.17733600 -0.80150600
H 4.43925200 1.06437100 4.53285900	H 6.47204200 -1.44767400 1.59810200	H -4.34938400 -4.40054700 -1.81477500
CP corrected structures		
C 1.318680 -0.437793 -1.719368	C 1.415246 0.430343 -0.880370	
C 3.157362 -0.782830 1.477754	C 2.992571 -1.158540 0.461886	
C -1.002964 -4.892437 1.959076	C -0.859971 -4.260842 2.623719	
C -0.175390 -3.813495 1.627432	C -0.470857 -2.922212 2.555211	

C -0.629126 -2.789104 0.798309	C -0.842157 -2.120234 1.475805	
C -1.931873 -2.828941 0.292381	C -1.609445 -2.674045 0.455506	
C -2.770876 -3.910377 0.661636	C -2.052781 -4.015343 0.563221	
C -2.308661 -4.939356 1.477716	C -1.666583 -4.813447 1.630090	
H -0.629491 -5.687644 2.596745	H -0.554389 -4.868855 3.474562	
H 0.838439 -3.778781 2.019109	H 0.127012 -2.496914 3.360660	
H 0.025042 -1.963862 0.543664	H -0.539750 -1.076879 1.426202	
H -2.963539 -5.762740 1.747558	H -2.010174 -5.842734 1.704195	
H -2.718021 -1.873764 -0.509348	C -2.258784 -2.050636 -0.723382	
H -4.070239 -2.450637 -0.590208	C -3.150503 -3.093134 -1.276842	
H -5.224312 -1.981699 -1.220082	C -4.005897 -3.059041 -2.370467	
C -6.390563 -2.738313 -1.144126	C -4.706993 -4.215433 -2.707675	
C -6.413728 -3.961311 -0.460891	C -4.545800 -5.393085 -1.974193	
C -5.264742 -4.440811 0.162264	C -3.681974 -5.437201 -0.881282	
C -4.098701 -3.681915 0.095476	C -2.990174 -4.284741 -0.537064	
H -5.221526 -1.043940 -1.767267	H -4.129079 -2.149611 -2.954185	
H -7.293779 -2.377996 -1.627170	H -5.387806 -4.202922 -3.557882	
H -7.332460 -4.538090 -0.420551	H -5.101809 -6.284803 -2.261220	
H -5.281135 -5.389993 0.690139	H -3.556244 -6.355061 -0.312042	
C -4.529394 3.020991 2.468408	C -5.737201 1.378501 2.132583	
C -4.074934 1.712981 2.271689	C -4.810048 0.348862 1.978950	
C -3.215486 1.399286 1.219600	C -3.686176 0.504945 1.166038	
C -2.801470 2.408380 0.343242	C -3.507900 1.706694 0.489725	
C -3.246361 3.734322 0.569275	C -4.431702 2.762699 0.684793	
C -4.109502 4.040448 1.617266	C -5.546468 2.602453 1.491881	
H -5.207249 3.241560 3.287281	H -6.607237 1.229898 2.770597	
H -4.402693 0.921889 2.939455	H -4.960145 -0.594031 2.503184	
H -2.886403 0.374395 1.094395	H -2.970029 -0.303997 1.078119	
H -4.445145 5.061616 1.773431	H -6.251229 3.418293 1.633245	
C -1.863465 2.417328 -0.799707	C -2.409295 2.193853 -0.384444	
C -1.775841 3.826826 -1.226147	C -2.717362 3.623026 -0.643139	
C -1.034635 4.424656 -2.245686	C -2.021779 4.575915 -1.373927	
C -1.137141 5.800461 -2.438991	C -2.556426 5.859855 -1.479634	
C -1.976825 6.579369 -1.632962	C -3.771652 6.182504 -0.873779	
C -2.723133 5.988823 -0.615528	C -4.477465 5.227336 -0.142721	
C -2.616788 4.615005 -0.415661	C -3.944084 3.952330 -0.031220	

H -0.389003 3.834393 -2.889306	H -1.075956 4.333994 -1.853337	
H -0.563402 6.273568 -3.230177	H -2.019475 6.621691 -2.043331	
H -2.049032 7.648640 -1.806209	H -4.171187 7.190770 -0.974143	
H -3.376368 6.592203 0.008156	H -5.424082 5.479000 0.329131	
C -1.098970 1.437381 -1.362427	C -1.323460 1.563639 -0.874350	
H -0.343494 1.777218 -2.071367	H -0.557000 2.171383 -1.363922	
C 4.387864 -2.657530 -3.643250	C 4.821190 0.892938 -3.351798	
C 3.104991 -3.231599 -3.622795	C 3.930747 -0.143406 -3.686582	
C 2.032830 -2.579207 -3.024886	C 2.785103 -0.377066 -2.942074	
C 2.242906 -1.336445 -2.409576	C 2.530524 0.416884 -1.815617	
C 3.542701 -0.777881 -2.412336	C 3.439736 1.460901 -1.477366	
C 4.609002 -1.425100 -3.039284	C 4.569946 1.705638 -2.257031	
H 5.202535 -3.175318 -4.139734	H 5.695215 1.071270 -3.976086	
H 2.943190 -4.193159 -4.100873	H 4.133183 -0.752336 -4.567756	
H 1.046079 -3.031972 -3.050446	H 2.080538 -1.148848 -3.244148	
H 5.598563 -0.975679 -3.055376	H 5.238625 2.528092 -2.011960	
C 2.136063 0.688729 -1.259593	C 1.644178 1.563857 0.003089	
C 1.863508 1.830397 -0.484243	C 0.910272 2.065420 1.088514	
C 2.894442 2.694611 -0.128654	C 1.424332 3.123603 1.819965	
C 4.218001 2.457805 -0.530654	C 2.669018 3.695146 1.504705	
C 4.513054 1.337099 -1.300267	C 3.412667 3.212461 0.438146	
C 3.486639 0.463626 -1.662154	C 2.904844 2.152303 -0.314256	
H 0.855212 2.066317 -0.168011	H -0.058122 1.642093 1.344292	
H 2.656023 3.584699 0.448246	H 0.848339 3.528516 2.651721	
H 5.003729 3.157309 -0.260869	H 3.039163 4.532765 2.093797	
H 5.531960 1.148278 -1.629116	H 4.368931 3.662680 0.179662	
C -0.024222 -0.767427 -1.561101	C 0.226077 -0.329781 -1.055722	
H -0.264356 -1.805343 -1.793675	H 0.334774 -1.350336 -1.434093	
C -2.414418 -0.634189 -1.000043	C -2.160095 -0.782941 -1.175237	
H -3.268242 -0.031201 -1.312611	H -2.919289 -0.412592 -1.871021	
C -1.143595 0.007794 -1.201472	C -1.061659 0.132010 -0.889289	
C 3.933290 0.185520 2.061714	C 4.254460 -0.561809 0.366415	
C 2.566013 0.251141 2.176393	C 3.458918 -0.271613 1.435102	
C 1.413610 0.871529 2.767194	C 3.287249 0.295121 2.741002	
C 0.052025 0.732666 2.097582	C 1.912443 0.148962 3.358297	
C 0.316442 -0.055525 3.314735	C 3.006808 -0.763880 3.833322	

H 1.611072 1.788327 3.310752	H 3.891926 1.163581 2.992214	
H 0.019552 0.222322 1.138785	H 1.136737 -0.258659 2.710411	
H -0.584799 1.610369 2.162482	H 1.584222 0.960398 4.010458	
H -0.122530 0.256564 4.257202	H 3.456500 -0.581573 4.812199	
H 0.475839 -1.125233 3.209075	H 2.990078 -1.808477 3.514871	
C 3.094082 -2.049081 0.797234	C 2.296455 -2.386386 0.171648	
C 4.337473 -2.553277 0.096419	C 2.723474 -3.128754 -1.083507	
C 3.967359 -3.161338 1.395580	C 3.190831 -3.637323 0.255190	
H 2.133109 -2.328734 0.384857	H 1.269928 -2.479906 0.515878	
H 5.183326 -1.872540 0.056752	H 3.452774 -2.630050 -1.723656	
H 4.155522 -3.106980 -0.819934	H 1.949776 -3.691042 -1.611652	
H 3.511364 -4.146558 1.405464	H 2.719475 -4.533342 0.666016	
H 4.549172 -2.914199 2.278917	H 4.237650 -3.491706 0.526163	
C 5.222433 0.716131 2.435263	C 5.563660 -0.488322 -0.216711	
C 5.308334 2.195292 2.755052	C 6.420269 0.673995 0.249074	
C 5.361313 1.220577 3.871374	C 6.712005 -0.701892 0.787402	
H 6.083012 0.241232 1.979345	H 5.672499 -0.847886 -1.237350	
H 4.401510 2.773542 2.605728	H 5.937440 1.402973 0.903454	
H 6.223620 2.690319 2.447007	H 7.108087 1.096865 -0.486035	
H 6.313725 1.018981 4.350990	H 7.593808 -1.228076 0.414604	
H 4.492786 1.119460 4.515421	H 6.436325 -0.930153 1.818560	

Table S2. Cartesian coordinates (in Å) of the optimized structures of the ion pair, ts and the covalent form in THF, using the continuum CPCM solvent model. The calculations have been performed at the M05-2X level with the 6-31+G*(C)/sto-3G(H) basis set.

ion pair	ts	covalent
C 1.28154600 -0.38033900 -1.74665700	C 1.54692100 0.20238800 -0.78662000	C -1.23105700 -1.10055400 0.54571600
C 3.34781300 -0.65972400 1.50014400	C 2.80769900 -1.36855100 0.34784600	C -1.59757900 -2.62183800 0.61397000
C -0.78697300 -5.02789500 1.82030900	C -1.57365800 -4.35161500 2.50408200	C 4.68538100 -2.06210400 -2.61825100
C 0.00190700 -3.91489900 1.50117500	C -0.89921300 -3.12781400 2.44200800	C 3.30984800 -1.84498300 -2.50977400
C -0.50631300 -2.87032100 0.73153500	C -1.10241500 -2.25178900 1.37475800	C 2.77022500 -1.21985500 -1.38447700
C -1.82832100 -2.92312100 0.27385200	C -1.98979000 -2.60704900 0.35790500	C 3.61799300 -0.79106600 -0.36477100
C -2.62760300 -4.03978400 0.63244500	C -2.70493800 -3.82198900 0.45536700	C 5.00947900 -0.99918200 -0.49276300
C -2.10981100 -5.09025600 1.38815100	C -2.49045700 -4.70136100 1.51277100	C 5.54585200 -1.63776400 -1.60541900
H -0.36891500 -5.83589700 2.41269700	H -1.39519700 -5.02370100 3.33772700	H 5.08636900 -2.55809000 -3.49659000
H 1.02685800 -3.86640900 1.85964300	H -0.20681100 -2.85614900 3.23379800	H 2.64787900 -2.17007600 -3.30668800
H 0.12267400 -2.02169900 0.49174800	H -0.57195000 -1.30594600 1.33627100	H 1.70073900 -1.06527100 -1.32049400
H -2.73368800 -5.93973700 1.65114900	H -3.04075700 -5.63532000 1.57736900	H 6.61676600 -1.79389700 -1.69195700
C -2.66611100 -1.95657000 -0.45581900	C -2.48418700 -1.85978600 -0.82179000	C 3.36668400 -0.03996000 0.89187800
C -4.00721200 -2.55857500 -0.50552000	C -3.56089300 -2.69653300 -1.39481800	C 4.70793100 0.17052800 1.49586500
C -5.19833900 -2.09218200 -1.06976000	C -4.37977900 -2.48666300 -2.50214500	C 5.07844300 0.80348300 2.67914000
C -6.34531300 -2.87740900 -0.97669300	C -5.31039300 -3.46734100 -2.84625800	C 6.43046000 0.85051100 3.02230700
C -6.31539400 -4.12691700 -0.34039700	C -5.41491000 -4.64969900 -2.10372300	C 7.39980600 0.26681500 2.19963000
C -5.13086300 -4.60481400 0.21636500	C -4.59209900 -4.86857000 -0.99891800	C 7.03278700 -0.37326100 1.01563500
C -3.98259800 -3.81840800 0.13177200	C -3.66872500 -3.88689000 -0.65007600	C 5.68485800 -0.41588600 0.67304400
H -5.23950500 -1.13478500 -1.58106200	H -4.30118600 -1.58034100 -3.09506200	H 4.34009000 1.25531500 3.33427500
H -7.27488900 -2.51907100 -1.40902900	H -5.95879400 -3.31342200 -3.70339500	H 6.73238800 1.34355200 3.94109800
H -7.21982200 -4.72494000 -0.28522100	H -6.14194400 -5.40269100 -2.39200800	H 8.44593300 0.31213900 2.48612400
H -5.10628400 -5.57362500 0.70729500	H -4.67375300 -5.78692000 -0.42506500	H 7.78582600 -0.82770000 0.37900900
C -4.60626000 2.85042300 2.58425300	C -5.39776300 2.02639400 2.22823400	C 1.17529400 3.59235200 -3.49700600
C -4.04624300 1.57812700 2.41124600	C -4.65119000 0.86367500 2.01649700	C 1.74062100 2.44563000 -2.93255300
C -3.20175100 1.30160000 1.33735400	C -3.54157500 0.86698500 1.16894400	C 1.37771400 2.02837000 -1.65028500
C -2.90460600 2.31186900 0.41371100	C -3.18680500 2.04854800 0.51784000	C 0.43754700 2.76641100 -0.94049000
C -3.44927400 3.60517700 0.61962600	C -3.92024000 3.23012100 0.77259100	C -0.11773500 3.93296000 -1.50657200
C -4.30184200 3.87324300 1.68880300	C -5.02782600 3.22346100 1.61401400	C 0.24305300 4.34911500 -2.78337100
H -5.26959300 3.04122000 3.42226700	H -6.25885100 2.00204000 2.88895300	H 1.46892600 3.90184400 -4.49533000
H -4.27497700 0.78879800 3.12144600	H -4.93223400 -0.05614000 2.52037400	H 2.47231800 1.87139300 -3.49336200

H	-2.78416100	0.30677200	1.23303400	H	-2.96725400	-0.04120900	1.03709200	H	1.83369200	1.14733700	-1.22018700
H	-4.71419400	4.86840900	1.82916300	H	-5.58655700	4.13517300	1.80322400	H	-0.18735400	5.24552900	-3.21946800
C	-2.00913300	2.36015700	-0.75834800	C	-2.05306400	2.37976200	-0.37407800	C	-0.13826000	2.56969800	0.40883000
C	-2.04172700	3.75734000	-1.22220900	C	-2.13204400	3.84327700	-0.58540100	C	-1.04596400	3.72424900	0.62196600
C	-1.38407800	4.38288600	-2.28485300	C	-1.30660200	4.70002400	-1.30989800	C	-1.84798100	4.05287500	1.70898000
C	-1.60256100	5.74047900	-2.51183800	C	-1.62487000	6.05764600	-1.36192600	C	-2.64468600	5.19692700	1.63439400
C	-2.47818300	6.47550400	-1.70001100	C	-2.75837400	6.55394400	-0.70777900	C	-2.64186900	5.99671200	0.48651500
C	-3.14395700	5.85836400	-0.64235700	C	-3.59030200	5.69940700	0.01655500	C	-1.84151300	5.66725100	-0.60899000
C	-2.92108000	4.50238500	-0.40752600	C	-3.26827700	4.34645900	0.07561100	C	-1.04554300	4.52868700	-0.53271700
H	-0.71444900	3.82739500	-2.93542100	H	-0.42765300	4.32927900	-1.82937300	H	-1.86830700	3.43208100	2.59972700
H	-1.09269200	6.23390900	-3.33385800	H	-0.98629800	6.73627600	-1.91901600	H	-3.27544700	5.46680500	2.47582300
H	-2.63981100	7.53037400	-1.89940700	H	-2.99127900	7.61275600	-0.76479300	H	-3.26991300	6.88134700	0.44735900
H	-3.82378800	6.42732100	-0.01454500	H	-4.46863400	6.08699300	0.52411900	H	-1.84479200	6.28811500	-1.49975400
C	-1.19337200	1.41630300	-1.32265200	C	-1.06938600	1.61146000	-0.89342900	C	0.00909700	1.55810900	1.27867100
H	-0.46028700	1.79285900	-2.03680600	H	-0.25047800	2.13902000	-1.38035600	H	-0.53008600	1.62920200	2.22204100
C	4.38276000	-2.45401300	-3.78466900	C	4.84002700	0.89191100	-3.37132000	C	-4.04160500	1.15087000	2.89891400
C	3.12199500	-3.07149100	-3.75549600	C	3.93665400	-0.11646000	-3.75465400	C	-3.12945100	0.35169500	3.59409400
C	2.04095200	-2.47080600	-3.11540600	C	2.81873000	-0.41102600	-2.98216700	C	-2.17677900	-0.40690000	2.90781100
C	2.22537200	-1.23992300	-2.47359300	C	2.60437400	0.30072200	-1.79359200	C	-2.14679800	-0.35509600	1.51954300
C	3.50228200	-0.63627100	-2.48873400	C	3.50718100	1.32846700	-1.42019700	C	-3.04515800	0.47036500	0.82632000
C	4.57708000	-1.22990900	-3.15089500	C	4.62218900	1.62298500	-2.20930100	C	-4.00034200	1.22171500	1.50631200
H	5.20682200	-2.93459600	-4.30298400	H	5.70139600	1.10849300	-3.99599800	H	-4.77388200	1.73552600	3.44763800
H	2.98345300	-4.02773200	-4.25143100	H	4.10915600	-0.66431000	-4.67669600	H	-3.15804500	0.32171800	4.67921800
H	1.07080600	-2.95926600	-3.12830500	H	2.11882200	-1.17756500	-3.30311300	H	-1.46089500	-1.00828800	3.45788100
H	5.55339800	-0.75270900	-3.16468400	H	5.31004700	2.41264600	-1.91829600	H	-4.68824500	1.86683800	0.96708900
C	2.07630800	0.76881000	-1.28008400	C	1.77879800	1.31435800	0.13240800	C	-1.66393500	-0.49072700	-0.78429800
C	1.78019500	1.87821600	-0.47192300	C	1.07643900	1.73695000	1.27097300	C	-1.16065200	-0.73099700	-2.05586700
C	2.79138100	2.76724200	-0.11578000	C	1.58609500	2.77495000	2.04037300	C	-1.72211900	-0.06078100	-3.14594000
C	4.11027300	2.58341700	-0.55424200	C	2.80339000	3.39562100	1.70619600	C	-2.77575200	0.84000500	-2.96280000
C	4.42705600	1.49071400	-1.35623000	C	3.51852700	2.98017200	0.58889500	C	-3.28828900	1.07833700	-1.68733900
C	3.42004500	0.59369300	-1.71285300	C	3.00450600	1.94409700	-0.19874400	C	-2.72805000	0.40406000	-0.60492000
H	0.77400900	2.06201500	-0.11590200	H	0.13494800	1.26896600	1.54546100	H	-0.35032900	-1.43651400	-2.21384500
H	2.54679600	3.62047900	0.51133000	H	1.03740800	3.10970700	2.91634800	H	-1.33231300	-0.23810600	-4.14390600
H	4.88234600	3.29058500	-0.26656200	H	3.18271700	4.20372300	2.32439700	H	-3.19674200	1.35780400	-3.81939200
H	5.44776700	1.33421700	-1.69509800	H	4.46120600	3.45541200	0.32969500	H	-4.10522900	1.78009400	-1.54406600
C	-0.03736500	-0.74910000	-1.56919900	C	0.27716400	-0.43200800	-1.04894300	C	0.24954300	-0.86531200	0.80587600

H	-0.25980400	-1.78321500	-1.83482700	H	0.30049700	-1.45814000	-1.41698200	H	0.89671000	-1.74042000	0.76109500
C	-2.41170600	-0.68738700	-0.91043800	C	-2.15161900	-0.63018300	-1.27604300	C	2.24409800	0.46301500	1.44733600
H	-3.29610900	-0.09556100	-1.15201200	H	-2.81723300	-0.17262200	-2.00918000	H	2.39439000	1.09904400	2.32004100
C	-1.17656200	-0.00685900	-1.15730700	C	-0.95918800	0.15485700	-0.95157700	C	0.82036100	0.32039600	1.09126000
C	4.08785000	0.34620700	2.07011500	C	4.14548000	-0.92677900	0.37738900	C	-2.84525100	-3.06775800	-0.08233200
C	2.72295100	0.32599000	2.22983600	C	3.39241400	-0.76389200	1.47926300	C	-1.73112500	-3.46477400	-0.62794500
C	1.53842200	0.86113300	2.84363400	C	3.28815700	-0.47083200	2.89511700	C	-1.01118600	-4.23623100	-1.63838800
C	0.19107700	0.62837900	2.17526500	C	1.92805800	-0.47568200	3.54532100	C	0.47919700	-4.06377500	-1.79929400
C	0.52424100	-0.15320100	3.38420900	C	2.83653400	-1.63031100	3.78265900	C	-0.04904500	-5.31209300	-1.16849100
H	1.67435500	1.78000500	3.40139600	H	4.04631900	0.19128900	3.29893700	H	-1.57771800	-4.46480100	-2.53697900
H	0.19836000	0.12824900	1.21163100	H	1.08171500	-0.61827200	2.88183400	H	0.96638500	-3.34763300	-1.14478200
H	-0.51064000	1.45280300	2.25616600	H	1.77163600	0.24374500	4.34244800	H	0.88558700	-4.11913100	-2.80442100
H	0.06788000	0.11382600	4.33167900	H	3.33739400	-1.71935800	4.74120600	H	-0.02467400	-6.23472900	-1.73982100
H	0.77414200	-1.20392600	3.26621100	H	2.61635700	-2.57091300	3.28497700	H	0.07323700	-5.42610100	-0.09545100
C	3.31301000	-1.91503500	0.79710800	C	2.11288700	-2.61788000	0.00306700	C	-1.19578500	-3.27218800	1.92896700
C	4.53953300	-2.36061300	0.02818200	C	2.49206600	-3.37003400	-1.24544400	C	-2.23785000	-3.63834900	2.95341100
C	4.25748900	-2.99796500	1.33531000	C	2.93580700	-3.88964700	0.08760300	C	-1.52401400	-4.71642900	2.18696700
H	2.34320300	-2.23153000	0.43470200	H	1.08731800	-2.69720900	0.34131800	H	-0.24322200	-2.93839800	2.33673700
H	5.35820300	-1.64984700	-0.04071800	H	3.24944600	-2.92064400	-1.88002700	H	-3.27327900	-3.43782900	2.68974700
H	4.33339600	-2.91593600	-0.88108000	H	1.69984500	-3.89349800	-1.77243300	H	-2.00436000	-3.50497500	4.00602900
H	3.84323600	-4.00096300	1.35465700	H	2.43861800	-4.76089000	0.50254800	H	-0.78759300	-5.31947600	2.70980400
H	4.87071300	-2.73270400	2.19098800	H	3.98680100	-3.79064300	0.34673400	H	-2.08521700	-5.24334600	1.41939200
C	5.35598000	0.94754000	2.38872100	C	5.45912100	-0.90778000	-0.24019800	C	-4.30066600	-2.92868200	-0.12259600
C	5.37492300	2.43560100	2.69879700	C	6.48173100	0.06678800	0.29257100	C	-4.90562300	-2.25064900	-1.33687000
C	5.52308700	1.47416800	3.81500400	C	6.63039200	-1.38332500	0.60839900	C	-5.08316500	-3.72464300	-1.13896100
H	6.22357500	0.51217300	1.90808200	H	5.48019800	-1.12832300	-1.30202100	H	-4.78234600	-2.72070500	0.82783600
H	4.43299900	2.96510100	2.59098300	H	6.14963800	0.73592800	1.08183500	H	-4.20537300	-1.91109900	-2.09503400
H	6.25123600	2.97065200	2.34764300	H	7.14920400	0.51005000	-0.43941800	H	-5.75642900	-1.59743200	-1.16636300
H	6.50481500	1.31922400	4.25020900	H	7.39700200	-1.95286500	0.09253700	H	-6.06082400	-4.09224200	-0.84288200
H	4.68668400	1.33067000	4.49222900	H	6.40462400	-1.71802700	1.61673300	H	-4.51328600	-4.39524900	-1.77642400

Table S3. Cartesian coordinates (in Å) of the optimized structures of the ion pair, ts and the covalent form in dichloromethane, using the continuum CPCM solvent model. The calculations have been performed at the M05-2X level with the 6-31+G*(C)/sto-3G(H) basis set.

ion pair				ts				covalent			
C	1.27955300	-0.37919900	-1.74691900	C	1.54885700	0.19797700	-0.78553300	C	-1.23320600	-1.10109400	0.54406100
C	3.35959200	-0.65959000	1.50315100	C	2.80502000	-1.37152000	0.34688900	C	-1.59891100	-2.62239200	0.61567900
C	-0.78525900	-5.04088900	1.80567500	C	-1.58517500	-4.35289300	2.50102500	C	4.68055800	-2.05893100	-2.62168200
C	0.00490700	-3.92868400	1.48680200	C	-0.90590100	-3.13174700	2.43919400	C	3.30533500	-1.83956900	-2.51338400
C	-0.50382400	-2.88053400	0.72238600	C	-1.10638500	-2.25425200	1.37261600	C	2.76635400	-1.21558700	-1.38712500
C	-1.82783900	-2.92888000	0.26967600	C	-1.99586700	-2.60543400	0.35619200	C	3.61452200	-0.79031800	-0.36623600
C	-2.62814200	-4.04504800	0.62786100	C	-2.71553600	-3.81773100	0.45328100	C	5.00572000	-1.00046400	-0.49413300
C	-2.10986100	-5.09901500	1.37840700	C	-2.50394100	-4.69854800	1.51006900	C	5.54144800	-1.63793900	-1.60777600
H	-0.36673900	-5.85157300	2.39406700	H	-1.40870900	-5.02620800	3.33409400	H	5.08099800	-2.55401400	-3.50078200
H	1.03135100	-3.88355300	1.84137200	H	-0.21165200	-2.86343300	3.23050200	H	2.64316900	-2.16200300	-3.31121800
H	0.12682300	-2.03304100	0.48294000	H	-0.57192700	-1.31067700	1.33436800	H	1.69712200	-1.05907200	-1.32335900
H	-2.73462800	-5.94792800	1.64117800	H	-3.05769700	-5.63048100	1.57437300	H	6.61211500	-1.79575600	-1.69427200
C	-2.66644500	-1.95853600	-0.45406900	C	-2.48789000	-1.85570100	-0.82293700	C	3.36398300	-0.04104500	0.89169400
C	-4.00885400	-2.55784500	-0.50090500	C	-3.56758300	-2.68842700	-1.39641700	C	4.70538700	0.16653400	1.49635300
C	-5.20121300	-2.08760800	-1.05959200	C	-4.38563400	-2.47520700	-2.50374300	C	5.07650000	0.79713000	2.68073200
C	-6.34944200	-2.87080100	-0.96449800	C	-5.31978700	-3.45243300	-2.84825300	C	6.42852400	0.84148100	3.02436400
C	-6.31975600	-4.12208200	-0.33159300	C	-5.42865500	-4.63462200	-2.10607100	C	7.39720200	0.25744800	2.20110000
C	-5.13411600	-4.60384500	0.21954100	C	-4.60669100	-4.85685900	-1.00125300	C	7.02957000	-0.38026200	1.01598300
C	-3.98451900	-3.81952500	0.13286200	C	-3.67976200	-3.87863000	-0.65207400	C	5.68165600	-0.42015900	0.67289600
H	-5.24246300	-1.12878100	-1.56824600	H	-4.30371700	-1.56904700	-3.09647500	H	4.33868800	1.24911900	3.33637100
H	-7.27983900	-2.50943800	-1.39254000	H	-5.96749800	-3.29590900	-3.70544700	H	6.73096200	1.33262900	3.94400500
H	-7.22518500	-4.71843300	-0.27470400	H	-6.15839400	-5.38488800	-2.39465800	H	8.44330700	0.30062200	2.48802000
H	-5.10971800	-5.57402300	0.70779700	H	-4.69168000	-5.77513200	-0.42776100	H	7.78209800	-0.83495400	0.37893100
C	-4.61061400	2.84972700	2.58548600	C	-5.39057600	2.03405200	2.23115200	C	1.18243600	3.60104300	-3.49137200
C	-4.04829800	1.57810000	2.41431100	C	-4.64613900	0.87003400	2.01872400	C	1.74782300	2.45411800	-2.92731200
C	-3.20334100	1.30149200	1.34083700	C	-3.53704700	0.87155600	1.17044700	C	1.38350700	2.03529900	-1.64591300
C	-2.90779700	2.31096700	0.41568100	C	-3.18064400	2.05267300	0.51936100	C	0.44145800	2.77164500	-0.93676200
C	-3.45457300	3.60375000	0.61993900	C	-3.91183400	3.23551600	0.77484200	C	-0.11351000	3.93869300	-1.50226800
C	-4.30778900	3.87182100	1.68869600	C	-5.01894000	3.23063600	1.61697600	C	0.24882100	4.35651500	-2.77812700
H	-5.27419000	3.04059300	3.42328900	H	-6.25125200	2.01107100	2.89245400	H	1.47714900	3.91172400	-4.48900200
H	-4.27545600	0.78951800	3.12585500	H	-4.92845000	-0.04932100	2.52275100	H	2.48068300	1.88101600	-3.48776700
H	-2.78388900	0.30728100	1.23797500	H	-2.96423000	-0.03753100	1.03809900	H	1.83972900	1.15423400	-1.21608200

H	-4.72170200	4.86652800	1.82777000	H	-5.57595900	4.14326200	1.80680900	H	-0.18142000	5.25321300	-3.21379900
C	-2.01240800	2.35910600	-0.75629200	C	-2.04677200	2.38206300	-0.37302700	C	-0.13665500	2.57273600	0.41126800
C	-2.04666200	3.75564800	-1.22161700	C	-2.12317700	3.84577800	-0.58390700	C	-1.04420800	3.72735200	0.62495100
C	-1.38955500	4.38106800	-2.28481400	C	-1.29649500	4.70123100	-1.30859600	C	-1.84702700	4.05507800	1.71168900
C	-1.60983300	5.73810600	-2.51333500	C	-1.61222600	6.05950100	-1.35995700	C	-2.64333500	5.19944800	1.63765000
C	-2.48679600	6.47287100	-1.70259400	C	-2.74449100	6.55770300	-0.70504300	C	-2.63936800	6.00050800	0.49062000
C	-3.15206800	5.85595200	-0.64449600	C	-3.57770700	5.70448600	0.01941100	C	-1.83815700	5.67206400	-0.60457200
C	-2.92740000	4.50047300	-0.40806700	C	-3.25818300	4.35087800	0.07784300	C	-1.04252100	4.53317800	-0.52878900
H	-0.71885900	3.82587700	-2.93455600	H	-0.41859500	4.32902100	-1.82882200	H	-1.86827400	3.43344900	2.60182500
H	-1.10028000	6.23136800	-3.33566100	H	-0.97270300	6.73713300	-1.91717700	H	-3.27470500	5.46859100	2.47885700
H	-2.64973100	7.52732200	-1.90314600	H	-2.97543600	7.61696900	-0.76156800	H	-3.26717400	6.88532800	0.45189400
H	-3.83282900	6.42468400	-0.01747600	H	-4.45505000	6.09359500	0.52752000	H	-1.84046900	6.29393300	-1.49463700
C	-1.19563400	1.41549100	-1.32011100	C	-1.06466700	1.61203900	-0.89283500	C	0.00749700	1.55884600	1.27905800
H	-0.46233500	1.79274400	-2.03367600	H	-0.24491400	2.13820000	-1.37986100	H	-0.53386400	1.62861900	2.22124700
C	4.38106600	-2.44212500	-3.79535500	C	4.83987200	0.88754400	-3.37318200	C	-4.04552100	1.15787100	2.88790900
C	3.12161200	-3.06212900	-3.76561200	C	3.93612500	-0.12069000	-3.75584600	C	-3.13604700	0.35832200	3.58619200
C	2.04057800	-2.46515500	-3.12189800	C	2.81869500	-0.41513900	-2.98248500	C	-2.18307000	-0.40273400	2.90306200
C	2.22395700	-1.23566900	-2.47730600	C	2.60529700	0.29679600	-1.79392700	C	-2.15011800	-0.35304800	1.51475000
C	3.49954400	-0.62944900	-2.49310600	C	3.50822100	1.32464900	-1.42140800	C	-3.04584100	0.47267700	0.81843500
C	4.57427200	-1.21927400	-3.15868300	C	4.62290000	1.61883600	-2.21108200	C	-4.00130000	1.22654000	1.49528300
H	5.20522500	-2.91989600	-4.31612100	H	5.70112600	1.10363400	-3.99818800	H	-4.77796900	1.74454300	3.43424500
H	2.98410400	-4.01746900	-4.26357100	H	4.10813900	-0.66891900	-4.67775200	H	-3.16692700	0.33006000	4.67129600
H	1.07138500	-2.95554300	-3.13401800	H	2.11885500	-1.18209100	-3.30257900	H	-1.46927100	-1.00433300	3.45557000
H	5.54974700	-0.74039400	-3.17264500	H	5.31129600	2.40813900	-1.92039900	H	-4.68727000	1.87174800	0.95368900
C	2.07324800	0.77028000	-1.27849600	C	1.78129600	1.31060600	0.13285400	C	-1.66398500	-0.49446200	-0.78811100
C	1.77631700	1.87657900	-0.46657100	C	1.08014600	1.73280000	1.27221900	C	-1.16055900	-0.73988800	-2.05871600
C	2.78652300	2.76647200	-0.10963800	C	1.59027200	2.77107100	2.04106900	C	-1.71950000	-0.07136000	-3.15115900
C	4.10488600	2.58633900	-0.55106800	C	2.80691500	3.39220400	1.70540000	C	-2.77107400	0.83258600	-2.97134800
C	4.42237600	1.49651800	-1.35678100	C	3.52103300	2.97685300	0.58734100	C	-3.28429000	1.07548300	-1.69700200
C	3.41635100	0.59866900	-1.71398900	C	3.00645300	1.94052900	-0.19960200	C	-2.72646000	0.40298100	-0.61216300
H	0.77049000	2.05691300	-0.10770800	H	0.13933200	1.26415100	1.54790400	H	-0.35226800	-1.44828300	-2.21416200
H	2.54178000	3.61696400	0.52110200	H	1.04275200	3.10530700	2.91797800	H	-1.32981000	-0.25301300	-4.14839800
H	4.87628500	3.29370000	-0.26209900	H	3.18686200	4.20023200	2.32332400	H	-3.19041600	1.34869900	-3.82976400
H	5.44290400	1.34253800	-1.69726900	H	4.46348200	3.45209200	0.32732700	H	-4.10025200	1.77894200	-1.55654700
C	-0.03863400	-0.74900500	-1.56988800	C	0.27785500	-0.43401600	-1.04853500	C	0.24713300	-0.86486100	0.80516200
H	-0.26124200	-1.78229200	-1.83858600	H	0.29943000	-1.46012800	-1.41671800	H	0.89450700	-1.73982000	0.76011400

C	-2.41212100	-0.68825400	-0.90618800	C	-2.15156100	-0.62695200	-1.27663000	C	2.24179200	0.46208700	1.44783200
H	-3.29701200	-0.09541800	-1.14346400	H	-2.81563000	-0.16745400	-2.00997200	H	2.39231200	1.09639800	2.32175800
C	-1.17795100	-0.00741800	-1.15526500	C	-0.95732800	0.15521500	-0.95146800	C	0.81800100	0.32072500	1.09115100
C	4.09433700	0.34923800	2.07504200	C	4.14372300	-0.93178000	0.37852200	C	-2.84510400	-3.07125500	-0.08137700
C	2.72974800	0.32055700	2.23576900	C	3.39044700	-0.77040200	1.48025200	C	-1.72975700	-3.46870000	-0.62424600
C	1.54190800	0.84782700	2.85008600	C	3.28603700	-0.48097600	2.89688300	C	-1.00756400	-4.24293100	-1.63092300
C	0.19688000	0.60958300	2.17902300	C	1.92564400	-0.48510200	3.54663600	C	0.48245100	-4.06746900	-1.79251500
C	0.53257300	-0.17308000	3.38673000	C	2.83209400	-1.64192000	3.78120500	C	-0.04270400	-5.31407400	-1.15579700
H	1.67232000	1.76603400	3.41023700	H	4.04533600	0.17854700	3.30284600	H	-1.57326600	-4.47719800	-2.52855300
H	0.20865800	0.11180300	1.21424100	H	1.07923800	-0.62467500	2.88258500	H	0.96773900	-3.34690300	-1.14145000
H	-0.50921200	1.43018300	2.26079800	H	1.77017700	0.23239400	4.34569900	H	0.88884600	-4.12644200	-2.79743500
H	0.07351800	0.08930700	4.33417900	H	3.33249000	-1.73422900	4.73968300	H	-0.01599300	-6.23936500	-1.72270400
H	0.78857300	-1.22209500	3.26663800	H	2.61048100	-2.58084000	3.28100200	H	0.07976500	-5.42256600	-0.08220100
C	3.33171800	-1.91331500	0.79715900	C	2.11077200	-2.62126400	0.00132000	C	-1.19820400	-3.26903600	1.93282900
C	4.55901600	-2.34754300	0.02267100	C	2.49159800	-3.37386200	-1.24642500	C	-2.24123400	-3.63496400	2.95639300
C	4.28704400	-2.98960200	1.32950500	C	2.93337900	-3.89294800	0.08756300	C	-1.52397700	-4.71332800	2.19351400
H	2.36311300	-2.23684400	0.43771800	H	1.08472600	-2.70038100	0.33818700	H	-0.24699900	-2.93244200	2.34149100
H	5.37160800	-1.63001600	-0.04809200	H	3.25000500	-2.92483000	-1.88003800	H	-3.27666100	-3.43718000	2.69061600
H	4.35373900	-2.90294000	-0.88669700	H	1.70017600	-3.89752200	-1.77442400	H	-2.00961600	-3.49872700	4.00905900
H	3.88093400	-3.99590400	1.34865300	H	2.43559800	-4.76405100	0.50209500	H	-0.78716800	-5.31371800	2.71885700
H	4.90119400	-2.72070600	2.18338000	H	3.98400100	-3.79386700	0.34820200	H	-2.08280700	-5.24305400	1.42614200
C	5.35901700	0.95778600	2.39302500	C	5.45765300	-0.91330000	-0.23851100	C	-4.30062600	-2.93378600	-0.12426500
C	5.36914400	2.44572300	2.70486300	C	6.48061800	0.06069200	0.29490500	C	-4.90428500	-2.25624800	-1.33951200
C	5.52451400	1.48395400	3.81969200	C	6.62845300	-1.38960200	0.61023500	C	-5.08052100	-3.73049500	-1.14199500
H	6.22875400	0.52820300	1.91108500	H	5.47910800	-1.13355000	-1.30037900	H	-4.78434900	-2.72639600	0.82526800
H	4.42382900	2.96956400	2.59907400	H	6.14864400	0.72961800	1.08440300	H	-4.20317200	-1.91605600	-2.09659600
H	6.24174900	2.98641100	2.35309700	H	7.14850400	0.50389300	-0.43675200	H	-5.75615600	-1.60403300	-1.17045100
H	6.50775900	1.33427200	4.25328400	H	7.39489800	-1.95931900	0.09432500	H	-6.05828900	-4.09914200	-0.84758300
H	4.68985200	1.33449000	4.49775800	H	6.40214500	-1.72454400	1.61836400	H	-4.50884600	-4.40032600	-1.77867200

Table S4. Cartesian coordinates (in Å) of the optimized structures of the ion pair, ts and the covalent form in EDC, using the continuum CPCM solvent model. The calculations have been performed at the M05-2X level with the 6-31+G*(C)/sto-3G(H) basis set.

ion pair				ts				covalent			
C	1.27804000	-0.38169600	-1.74710800	C	1.54939600	0.19654400	-0.78423600	C	-1.23488500	-1.10140300	0.54291000
C	3.36732700	-0.66036100	1.50836400	C	2.80414900	-1.37242500	0.34530100	C	-1.60017300	-2.62266400	0.61646500
C	-0.79252800	-5.05027600	1.79692200	C	-1.58942100	-4.35297900	2.49971500	C	4.67688200	-2.05673300	-2.62413900
C	0.00056200	-3.94018700	1.47784400	C	-0.90810900	-3.13298300	2.43759700	C	3.30189700	-1.83575300	-2.51587800
C	-0.50656700	-2.88890000	0.71667400	C	-1.10759300	-2.25512700	1.37111900	C	2.76342300	-1.21275100	-1.38881800
C	-1.83208500	-2.93181800	0.26759900	C	-1.99810600	-2.60479300	0.35506700	C	3.61191600	-0.79015700	-0.36705900
C	-2.63510000	-4.04606000	0.62585500	C	-2.71962600	-3.81596700	0.45237700	C	5.00289600	-1.00179200	-0.49496500
C	-2.11843500	-5.10318700	1.37309000	C	-2.50914300	-4.69714300	1.50910100	C	5.53810600	-1.63831900	-1.60942500
H	-0.37513600	-5.86337900	2.38277900	H	-1.41367000	-5.02661500	3.33266900	H	5.07689400	-2.55103800	-3.50387200
H	1.02805600	-3.89920900	1.82981300	H	-0.21297300	-2.86592100	3.22854100	H	2.63953800	-2.15615600	-3.31437100
H	0.12661600	-2.04337900	0.47692600	H	-0.57149900	-1.31248700	1.33274400	H	1.69437400	-1.05488200	-1.32515700
H	-2.74537700	-5.95046200	1.63597700	H	-3.06432500	-5.62821400	1.57355800	H	6.60859200	-1.79733100	-1.69595000
C	-2.66949000	-1.95749900	-0.45220400	C	-2.48919900	-1.85440700	-0.82409300	C	3.36200200	-0.04234300	0.89188400
C	-4.01388700	-2.55248800	-0.49677800	C	-3.57015900	-2.68566200	-1.39745100	C	4.70354000	0.16303000	1.49700200
C	-5.20625600	-2.07750500	-1.05158000	C	-4.38792200	-2.47139200	-2.50479600	C	5.07513500	0.79177900	2.68222800
C	-6.35678500	-2.85713500	-0.95475200	C	-5.32359200	-3.44728200	-2.84908400	C	6.42716800	0.83413000	3.02616300
C	-6.32950600	-4.10954000	-0.32392500	C	-5.43424400	-4.62914000	-2.10663300	C	7.39532100	0.24992300	2.20237900
C	-5.14395600	-4.59606300	0.22326000	C	-4.61256600	-4.85242900	-1.00178200	C	7.02719500	-0.38594300	1.01640000
C	-3.99201700	-3.81534100	0.13482000	C	-3.68412000	-3.87553600	-0.65285900	C	5.67928600	-0.42380200	0.67298700
H	-5.24582600	-1.11774300	-1.55862700	H	-4.30465000	-1.56550300	-3.09776200	H	4.33773400	1.24381600	3.33830000
H	-7.28711500	-2.49205700	-1.37977600	H	-5.97106600	-3.28996500	-3.70631300	H	6.73000900	1.32380200	3.94646000
H	-7.23671600	-4.70304000	-0.26557000	H	-6.16514100	-5.37835500	-2.39503500	H	8.44141900	0.29148800	2.48957500
H	-5.12147700	-5.56711600	0.70988100	H	-4.69892300	-5.77046900	-0.42812300	H	7.77932500	-0.84075700	0.37896500
C	-4.60722100	2.85414600	2.58746800	C	-5.38738900	2.03626100	2.23214600	C	1.18937300	3.60766200	-3.48647700
C	-4.04444200	1.58247000	2.41779500	C	-4.64386600	0.87173300	2.01913100	C	1.75469200	2.46059900	-2.92259200
C	-3.20025200	1.30457500	1.34406700	C	-3.53506800	0.87267300	1.17044900	C	1.38884700	2.04048400	-1.64202500
C	-2.90586600	2.31278700	0.41709300	C	-3.17804300	2.05373800	0.51957800	C	0.44509000	2.77545500	-0.93366100
C	-3.45290000	3.60573500	0.61995100	C	-3.90825700	3.23707100	0.77567600	C	-0.10950000	3.94289200	-1.49882100
C	-4.30544200	3.87505500	1.68897600	C	-5.01509500	3.23277300	1.61820300	C	0.25442200	4.36205500	-2.77381700
H	-5.27019300	3.04599600	3.42552400	H	-6.24783100	2.01372000	2.89376600	H	1.48524000	3.91934800	-4.48344800
H	-4.27058100	0.79496100	3.13085800	H	-4.92666000	-0.04751900	2.52308700	H	2.48868100	1.88843800	-3.48253100
H	-2.78032000	0.31043600	1.24235800	H	-2.96286800	-0.03674700	1.03766400	H	1.84506000	1.15934900	-1.21229100

H	-4.71957300	4.86982100	1.82698300	H	-5.57138500	4.14573700	1.80853500	H	-0.17559700	5.25896800	-3.20926100
C	-2.01142500	2.35947200	-0.75555300	C	-2.04417900	2.38253000	-0.37302100	C	-0.13520900	2.57476700	0.41319000
C	-2.04619900	3.75529800	-1.22272800	C	-2.11949400	3.84637900	-0.58340100	C	-1.04287700	3.72930200	0.62699500
C	-1.38999900	4.37938200	-2.28733200	C	-1.29233700	4.70142700	-1.30806500	C	-1.84684900	4.05610100	1.71319000
C	-1.61077600	5.73603900	-2.51765900	C	-1.60696500	6.05999600	-1.35880800	C	-2.64299900	5.20059500	1.63932900
C	-2.48739900	6.47177800	-1.70737500	C	-2.73864300	6.55887800	-0.70336100	C	-2.63775700	6.00274200	0.49303700
C	-3.15179800	5.85622700	-0.64791800	C	-3.57237100	5.70607600	0.02102000	C	-1.83534800	5.67530100	-0.60158200
C	-2.92664100	4.50111600	-0.40965800	C	-3.25392600	4.35216600	0.07886000	C	-1.03984100	4.53628300	-0.52594600
H	-0.71957000	3.82344700	-2.93672100	H	-0.41494600	4.32870100	-1.82879400	H	-1.86901700	3.43377600	2.60281200
H	-1.10187400	6.22825900	-3.34101600	H	-0.96707600	6.73732000	-1.91599000	H	-3.27525600	5.46899400	2.48010700
H	-2.65070400	7.52590800	-1.90931000	H	-2.96873700	7.61835300	-0.75941500	H	-3.26549200	6.88761600	0.45445400
H	-3.83225300	6.42572900	-0.02125900	H	-4.44924200	6.09574400	0.52951400	H	-1.83661800	6.29802700	-1.49104900
C	-1.19517100	1.41494600	-1.31893600	C	-1.06277700	1.61189000	-0.89323600	C	0.00641100	1.55918800	1.27947900
H	-0.46177300	1.79141000	-2.03282300	H	-0.24269900	2.13758500	-1.38023300	H	-0.53690500	1.62785500	2.22059900
C	4.37678000	-2.44428300	-3.80011700	C	4.83957100	0.89361100	-3.37098900	C	-4.04869700	1.16207800	2.88062000
C	3.11732800	-3.06412600	-3.76919900	C	3.93688300	-0.11496200	-3.75520900	C	-3.14104500	0.36221300	3.58092600
C	2.03713100	-2.46725300	-3.12387300	C	2.81971700	-0.41173500	-2.98229500	C	-2.18772100	-0.40027100	2.89987200
C	2.22155700	-1.23818900	-2.47892300	C	2.60553300	0.29831500	-1.79278000	C	-2.15261600	-0.35173500	1.51155400
C	3.49709400	-0.63202400	-2.49609600	C	3.50727800	1.32663600	-1.41875000	C	-3.04646800	0.47432900	0.81323400
C	4.57093100	-1.22165200	-3.16320400	C	4.62179800	1.62303900	-2.20783900	C	-4.00228200	1.22962200	1.48800500
H	5.20033400	-2.92203100	-4.32186400	H	5.70081300	1.11129000	-3.99545600	H	-4.78142300	1.74987000	3.42537500
H	2.97912700	-4.01929000	-4.26730100	H	4.10964300	-0.66183600	-4.67777500	H	-3.17359900	0.33485300	4.66600300
H	1.06788600	-2.95758300	-3.13489700	H	2.12085700	-1.17917200	-3.30334700	H	-1.47535300	-1.00207000	3.45398200
H	5.54651200	-0.74302900	-3.17792700	H	5.30952600	2.41243700	-1.91585200	H	-4.68692000	1.87495500	0.94486800
C	2.07271400	0.76740600	-1.27882900	C	1.78038500	1.30851700	0.13546900	C	-1.66408300	-0.49660600	-0.79062700
C	1.77688600	1.87301000	-0.46567200	C	1.07855400	1.72841100	1.27520700	C	-1.16047800	-0.74527500	-2.06058200
C	2.78760200	2.76281400	-0.10984300	C	1.58720600	2.76648300	2.04535300	C	-1.71754100	-0.07757500	-3.15451900
C	4.10520500	2.58303400	-0.55358400	C	2.80305400	3.38961600	1.71050600	C	-2.76759000	0.82861600	-2.97683700
C	4.42160700	1.49361100	-1.36031000	C	3.51786100	2.97650500	0.59201600	C	-3.28136700	1.07434100	-1.70324500
C	3.41505700	0.59589600	-1.71629800	C	3.00470700	1.94038400	-0.19613800	C	-2.72540300	0.40265800	-0.61689900
H	0.77164700	2.05269300	-0.10482200	H	0.13836400	1.25811400	1.55021900	H	-0.35367600	-1.45572400	-2.21436200
H	2.54402400	3.61267500	0.52218500	H	1.03929400	3.09880400	2.92274700	H	-1.32787600	-0.26197000	-4.15126700
H	4.87704300	3.29015800	-0.26523800	H	3.18197700	4.19729500	2.32951800	H	-3.18561700	1.34390200	-3.83639100
H	5.44168900	1.33973100	-1.70213000	H	4.45976900	3.45320800	0.33275900	H	-4.09654800	1.77905700	-1.56459000
C	-0.04006000	-0.75051000	-1.56950900	C	0.27829200	-0.43500500	-1.04877200	C	0.24527900	-0.86468300	0.80482400
H	-0.26403500	-1.78319200	-1.83936100	H	0.29970900	-1.46106300	-1.41706800	H	0.89268600	-1.73962400	0.75958500

C	-2.41302100	-0.68693900	-0.90272700	C	-2.15134200	-0.62616800	-1.27797700	C	2.24014200	0.46099200	1.44852100
H	-3.29727800	-0.09199500	-1.13705000	H	-2.81461500	-0.16618500	-2.01173900	H	2.39086300	1.09398800	2.32337100
C	-1.17850200	-0.00775600	-1.15345300	C	-0.95649300	0.15496000	-0.95233900	C	0.81632800	0.32072500	1.09132100
C	4.10102200	0.35071700	2.07780100	C	4.14340700	-0.93378800	0.37671800	C	-2.84524100	-3.07332000	-0.08145000
C	2.73673600	0.31948600	2.24047200	C	3.39125300	-0.77349200	1.47921400	C	-1.72897800	-3.47105100	-0.62226900
C	1.54774300	0.84464200	2.85442600	C	3.28834600	-0.48665000	2.89650400	C	-1.00503900	-4.24671600	-1.62658100
C	0.20401700	0.60246100	2.18219000	C	1.92857800	-0.49097100	3.54762000	C	0.48484900	-4.06952000	-1.78779600
C	0.54066800	-0.17837800	3.39092700	C	2.83440300	-1.64892200	3.77904500	C	-0.03888100	-5.31526700	-1.14825300
H	1.67566500	1.76357200	3.41393600	H	4.04864000	0.17137300	3.30304400	H	-1.56984200	-4.48402300	-2.52397100
H	0.21846400	0.10403100	1.21778900	H	1.08136100	-0.62867900	2.88421000	H	0.96872800	-3.34657300	-1.13831500
H	-0.50432700	1.42124500	2.26264900	H	1.77446000	0.22502800	4.34828800	H	0.89175900	-4.13033000	-2.79239900
H	0.07994800	0.08375100	4.33763300	H	3.33570800	-1.74337400	4.73683900	H	-0.01056400	-6.24192400	-1.71284200
H	0.79965500	-1.22680400	3.27211100	H	2.61159300	-2.58671900	3.27727600	H	0.08318100	-5.42088500	-0.07432400
C	3.33882800	-1.91448700	0.80335100	C	2.11024300	-2.62246700	-0.00042300	C	-1.20063900	-3.26707100	1.93508000
C	4.56200900	-2.34538000	0.02020000	C	2.49025900	-3.37396900	-1.24911100	C	-2.24461400	-3.63255200	2.95784400
C	4.30126100	-2.98764300	1.32909600	C	2.93379200	-3.89359900	0.08410300	C	-1.52532400	-4.71129600	2.19739900
H	2.36846600	-2.24115400	0.45165900	H	1.08462000	-2.70275500	0.33755400	H	-0.25030100	-2.92893400	2.34449900
H	5.37178500	-1.62529600	-0.05636800	H	3.24779700	-2.92408300	-1.88315800	H	-3.27991400	-3.43619900	2.69048100
H	4.35198000	-2.90167000	-0.88751400	H	1.69871100	-3.89787300	-1.77667900	H	-2.01441000	-3.49457400	4.01059500
H	3.89813400	-3.99506000	1.35163500	H	2.43707600	-4.76534900	0.49854200	H	-0.78857100	-5.31021100	2.72450300
H	4.92066100	-2.71644100	2.17843300	H	3.98460700	-3.79393500	0.34373700	H	-2.08264600	-5.24264900	1.43005400
C	5.36487500	0.96227400	2.39278500	C	5.45662700	-0.91591000	-0.24191900	C	-4.30081800	-2.93681900	-0.12642800
C	5.37224600	2.45068500	2.70295600	C	6.48275000	0.05381900	0.29317200	C	-4.90327200	-2.25930900	-1.34231500
C	5.53161900	1.49058000	3.81857800	C	6.62757200	-1.39791700	0.60338400	C	-5.07870400	-3.73376700	-1.14546500
H	6.23470800	0.53390300	1.90996600	H	5.47610500	-1.13277900	-1.30451900	H	-4.78610100	-2.73004300	0.82245000
H	4.42551800	2.97220000	2.59832800	H	6.15389800	0.72075700	1.08566000	H	-4.20140800	-1.91847700	-2.09841600
H	6.24307000	2.99279200	2.34897600	H	7.15059200	0.49787000	-0.43801500	H	-5.75590500	-1.60781700	-1.17428100
H	6.51598800	1.34338200	4.25044900	H	7.39179700	-1.96778200	0.08435300	H	-6.05663000	-4.10318100	-0.85254200
H	4.69845100	1.34002500	4.49822700	H	6.40197900	-1.73561900	1.61074900	H	-4.50567100	-4.40295100	-1.78160700

Table S5. Cartesian coordinates (in Å) of the optimized structures of the ion pair, ts and the covalent form in acetonitrile, using the continuum CPCM solvent model. The calculations have been performed at the M05-2X level with the 6-31+G*(C)/sto-3G(H) basis set.

ion pair	ts	covalent
C 1.26913300 -0.38434700 -1.74502600	C 1.55442000 0.18280200 -0.78002600	C -1.24289100 -1.09941600 0.53985400
C 3.41040700 -0.65449200 1.52985300	C 2.79635700 -1.38153300 0.34191900	C -1.61100600 -2.61970400 0.61944700
C -0.79119500 -5.12312200 1.71682200	C -1.62260500 -4.35714300 2.48976000	C 4.66500100 -2.06323300 -2.62900900
C 0.00910700 -4.01608300 1.40470300	C -0.92779900 -3.14470300 2.42877100	C 3.29077400 -1.83698000 -2.52090100
C -0.49911200 -2.94298800 0.67540200	C -1.12006000 -2.26218000 1.36476500	C 2.75423800 -1.21473500 -1.39242800
C -1.83396100 -2.96068100 0.25123800	C -2.01677400 -2.59971200 0.35007100	C 3.60394900 -0.79846400 -0.36896700
C -2.64320200 -4.07286900 0.60298400	C -2.75098600 -3.80334900 0.44595400	C 4.99418600 -1.01539100 -0.49676400
C -2.12557700 -5.15141500 1.31846200	C -2.54823400 -4.68914300 1.50036700	C 5.52751900 -1.65104200 -1.61273600
H -0.37236300 -5.95290500 2.27778100	H -1.45210900 -5.03472700 3.32058000	H 5.06341300 -2.55690100 -3.50982300
H 1.04352200 -3.99480900 1.73731400	H -0.22720400 -2.88759500 3.21817300	H 2.62756000 -2.15277200 -3.32052400
H 0.14211300 -2.10173000 0.44138200	H -0.57322900 -1.32572800 1.32763700	H 1.68575300 -1.05283200 -1.32907300
H -2.75787500 -5.99628900 1.57632300	H -3.11306400 -5.61447200 1.56374200	H 6.59737600 -1.81425400 -1.69919200
C -2.67348800 -1.96450800 -0.43574200	C -2.50113500 -1.84198900 -0.82725600	C 3.35638400 -0.05310300 0.89197100
C -4.02520300 -2.54366600 -0.46609000	C -3.59083400 -2.66135000 -1.40179600	C 4.69846300 0.14519200 1.49843300
C -5.22298200 -2.04563300 -0.98926800	C -4.40651300 -2.43700500 -2.50874900	C 5.07185000 0.76925400 2.68568100
C -6.38053100 -2.81379300 -0.88382200	C -5.35244600 -3.40271800 -2.85421800	C 6.42397300 0.80511000 3.03043000
C -6.35586500 -4.07756100 -0.27560900	C -5.47532600 -4.58428900 -2.11321200	C 7.39022800 0.21910800 2.20555100
C -5.16555200 -4.58720100 0.23948000	C -4.65575000 -4.81771600 -1.00871400	C 7.02026900 -0.41210300 1.01754100
C -4.00633500 -3.81797900 0.14255500	C -3.71702900 -3.85097600 -0.65874700	C 5.67231600 -0.44338200 0.67325900
H -5.26190100 -1.07665600 -1.47871000	H -4.31386200 -1.53144300 -3.10088200	H 4.33599500 1.22234900 3.34277600
H -7.31448300 -2.43057100 -1.28422200	H -5.99818100 -3.23758700 -3.71130400	H 6.72829200 1.29091700 3.95229900
H -7.26866900 -4.66166600 -0.20994900	H -6.21403500 -5.32547300 -2.40252400	H 8.43631400 0.25548200 2.49352400
H -5.14505400 -5.56706200 0.70829900	H -4.75152200 -5.73570300 -0.43646400	H 7.77094500 -0.86832900 0.37938800
C -4.61384000 2.86794700 2.59439300	C -5.36317900 2.05871900 2.24375800	C 1.21732600 3.61923300 -3.47593000
C -4.04349100 1.59785800 2.43600000	C -4.62590300 0.89050100 2.02835500	C 1.77724900 2.46898400 -2.91291400
C -3.19995300 1.31445200 1.36331800	C -3.51950100 0.88617000 1.17645000	C 1.40575300 2.04687400 -1.63449100
C -2.91298500 2.31537200 0.42568100	C -3.15855800 2.06579400 0.52491400	C 0.46137500 2.78277300 -0.92762000
C -3.46687400 3.60744700 0.61777900	C -3.88213600 3.25275000 0.78353500	C -0.08718700 3.95380600 -1.49163700
C -4.31939600 3.88196600 1.68581400	C -4.98673400 3.25370400 1.62922100	C 0.28243300 4.37497100 -2.76448100
H -5.27601500 3.06401200 3.43209700	H -6.22171000 2.04024600 2.90797800	H 1.51730300 3.93231100 -4.47122800
H -4.26283600 0.81645100 3.15787500	H -4.91169000 -0.02726600 2.53338100	H 2.51111500 1.89588900 -3.47208000
H -2.77389000 0.32198100 1.27056200	H -2.95165300 -0.02576200 1.04198100	H 1.85719200 1.16283000 -1.20553000
H -4.73863500 4.87573500 1.81544100	H -5.53795400 4.16928600 1.82169800	H -0.14315500 5.27435800 -3.19918400
C -2.02141100 2.35584000 -0.74870300	C -2.02517500 2.38918300 -0.37016500	C -0.12525600 2.57989800 0.41628000
C -2.06318500 3.74693100 -1.22786400	C -2.09331000 3.85354100 -0.57950500	C -1.02839300 3.73796100 0.63054400
C -1.41188500 4.36495700 -2.29947300	C -1.26336500 4.70478200 -1.30571200	C -1.83404700 4.06555200 1.71541700
C -1.63983300 5.71830300 -2.54177400	C -1.57092300 6.06513800 -1.35464400	C -2.62462500 5.21398100 1.64230800
C -2.51916300 6.45719600 -1.73686000	C -2.69849700 6.56951100 -0.69612600	C -2.61234500 6.01940000 0.49821900

C	-3.17888800	5.84796700	-0.67084300	C	-3.53515400	5.72061500	0.02961000	C	-1.80827500	5.69133500	-0.59504100
C	-2.94661600	4.49600400	-0.42042100	C	-3.22366000	4.36489500	0.08577100	C	-1.01830800	4.54826900	-0.52010700
H	-0.73942500	3.80676700	-2.94486100	H	-0.38945900	4.32784300	-1.82935400	H	-1.86162600	3.44114600	2.60342300
H	-1.13444700	6.20563700	-3.37021800	H	-0.92892900	6.73950800	-1.91299900	H	-3.25813300	5.48294600	2.48195800
H	-2.68784700	7.50867800	-1.94800700	H	-2.92315900	7.63021000	-0.75090400	H	-3.23595500	6.90720600	0.46031700
H	-3.86115300	6.41984400	-0.04828400	H	-4.40877500	6.11466500	0.54032200	H	-1.80404500	6.31656400	-1.48274200
C	-1.20283800	1.40938700	-1.30772800	C	-1.04882400	1.61331000	-0.89218800	C	0.00681800	1.55984600	1.27898200
H	-0.47006300	1.78514100	-2.02263600	H	-0.22654800	2.13458200	-1.38028200	H	-0.54055400	1.62777000	2.21775400
C	4.36564200	-2.42443900	-3.82374800	C	4.83650700	0.88407900	-3.37682000	C	-4.05382400	1.18353300	2.86221700
C	3.11007600	-3.05134800	-3.78712200	C	3.93301500	-0.12421100	-3.75942500	C	-3.15166000	0.38186600	3.56764200
C	2.03080100	-2.46229300	-3.13258100	C	2.81793900	-0.42192500	-2.98350400	C	-2.19963400	-0.38689400	2.89180400
C	2.21326800	-1.23468700	-2.48510400	C	2.60689600	0.28755900	-1.79332800	C	-2.16026400	-0.34280400	1.50337400
C	3.48477400	-0.62112800	-2.50842700	C	3.50861000	1.31640300	-1.42155500	C	-3.04844400	0.48508400	0.79994500
C	4.55749400	-1.20262500	-3.18407800	C	4.62158600	1.61305600	-2.21268300	C	-4.00310700	1.24657400	1.46949500
H	5.18888400	-2.89652600	-4.35113400	H	5.69701000	1.10120700	-4.00249700	H	-4.78547800	1.77624900	3.40305400
H	2.97426500	-4.00613200	-4.28660000	H	4.10413200	-0.67138300	-4.68211900	H	-3.18731900	0.35820800	4.65270000
H	1.06437500	-2.95834800	-3.13771000	H	2.11952100	-1.19071200	-3.30225400	H	-1.49156000	-0.98990600	3.45004300
H	5.53072500	-0.71949200	-3.20222600	H	5.31072900	2.40144000	-1.92134600	H	-4.68382700	1.89278100	0.92242600
C	2.06206200	0.76671000	-1.27556600	C	1.78666000	1.29644400	0.13825100	C	-1.66750400	-0.49924700	-0.79726300
C	1.76542600	1.86467600	-0.45323100	C	1.08840800	1.71427400	1.28065300	C	-1.16458500	-0.75778400	-2.06567900
C	2.77356100	2.75915500	-0.10103500	C	1.59826000	2.75281000	2.04977100	C	-1.71693400	-0.09257700	-3.16368500
C	4.08806400	2.59025200	-0.55747200	C	2.81193500	3.37789500	1.71074700	C	-2.76188300	0.82075800	-2.99151200
C	4.40520800	1.50670800	-1.37212200	C	3.52356000	2.96618800	0.58949300	C	-3.27568000	1.07567700	-1.71961600
C	3.40128400	0.60454500	-1.72370700	C	3.00894500	1.92968100	-0.19718700	C	-2.72437400	0.40647500	-0.62920000
H	0.76249000	2.03321800	-0.08055900	H	0.15048200	1.24145600	1.55912400	H	-0.36270700	-1.47466100	-2.21530300
H	2.53113600	3.60236100	0.54020600	H	1.05396300	3.08273900	2.93034100	H	-1.32854700	-0.28566700	-4.15930100
H	4.85815200	3.29967900	-0.27025900	H	3.19264700	4.18494300	2.32951000	H	-3.17708500	1.33308300	-3.85422100
H	5.42391900	1.35983300	-1.72075800	H	4.46458400	3.44336300	0.32796900	H	-4.08827900	1.78429300	-1.58569900
C	-0.04643600	-0.75449800	-1.56584400	C	0.27963300	-0.44175200	-1.04678000	C	0.23755200	-0.86523000	0.80313100
H	-0.27311000	-1.78414400	-1.84462500	H	0.29581600	-1.46813100	-1.41434700	H	0.88269900	-1.74186700	0.75811100
C	-2.41452100	-0.68968800	-0.87495000	C	-2.15180900	-0.61658600	-1.27982400	C	2.23609200	0.45296700	1.44941400
H	-3.29958400	-0.08821300	-1.08842300	H	-2.81018000	-0.15092000	-2.01446600	H	2.38827700	1.08249900	2.32652900
C	-1.18351200	-0.01181700	-1.13887900	C	-0.95141300	0.15566600	-0.95220800	C	0.81200100	0.31836500	1.09055300
C	4.13209100	0.36953200	2.09238200	C	4.13856100	-0.94925800	0.37823900	C	-2.85415400	-3.07263800	-0.08045100
C	2.76929900	0.31818000	2.26136600	C	3.38701300	-0.79346600	1.48102300	C	-1.73636700	-3.47397200	-0.61559000
C	1.57166000	0.82765800	2.87230900	C	3.28536500	-0.51676500	2.90053600	C	-1.00944400	-4.25587100	-1.61284900
C	0.23462400	0.55262400	2.19986600	C	1.92582500	-0.51917800	3.55254200	C	0.48030100	-4.07645300	-1.77376300
C	0.58488500	-0.20947100	3.41744400	C	2.82653900	-1.68293700	3.77505800	C	-0.04125600	-5.31825100	-1.12483400
H	1.68211000	1.75378600	3.42347500	H	4.04930100	0.13406900	3.31188500	H	-1.57249700	-4.50171300	-2.50900500
H	0.26273200	0.04609300	1.23996600	H	1.07733200	-0.64906900	2.88917300	H	0.96145800	-3.34703500	-1.12951000
H	-0.49000700	1.35787500	2.27151900	H	1.77556600	0.19168300	4.35854500	H	0.88807100	-4.14398700	-2.77758600
H	0.11724200	0.05352700	4.36047300	H	3.32817300	-1.78624000	4.73174900	H	-0.01034000	-6.24916600	-1.68222200

H	0.86661400	-1.25326800	3.31107900	H	2.59896100	-2.61609800	3.26684300	H	0.07982400	-5.41505000	-0.04995400
C	3.39345300	-1.90859000	0.82524400	C	2.10422700	-2.63317700	-0.00503800	C	-1.21560400	-3.25818000	1.94218900
C	4.60626300	-2.30916600	0.00887900	C	2.48651100	-3.38489900	-1.25298700	C	-2.26262700	-3.62056200	2.96295600
C	4.39495900	-2.95894200	1.32247800	C	2.92837400	-3.90331700	0.08144800	C	-1.53935600	-4.70164100	2.20945900
H	2.42206700	-2.25806100	0.49940600	H	1.07821000	-2.71464300	0.33161500	H	-0.26715200	-2.91721900	2.35364500
H	5.39597300	-1.56944400	-0.08681800	H	3.24504600	-2.93506500	-1.88587600	H	-3.29733400	-3.42684900	2.69125600
H	4.38557600	-2.86887100	-0.89410600	H	1.69643600	-3.90984200	-1.78174700	H	-2.03583700	-3.47760000	4.01578200
H	4.01603100	-3.97540700	1.35402000	H	2.43190500	-4.77537400	0.49552800	H	-0.80347400	-5.29726700	2.74149000
H	5.02976000	-2.67356400	2.15562700	H	3.97868600	-3.80252800	0.34274200	H	-2.09322200	-5.23694500	1.44234700
C	5.38749800	1.00296900	2.39549600	C	5.45195800	-0.93253700	-0.24018700	C	-4.30974500	-2.93679400	-0.13059500
C	5.37233800	2.49305600	2.69952700	C	6.47936700	0.03581700	0.29575700	C	-4.90853000	-2.25936800	-1.34845700
C	5.55546000	1.54074200	3.81779900	C	6.62249300	-1.41635800	0.60444900	C	-5.08339100	-3.73410600	-1.15250500
H	6.26092100	0.58635000	1.90900200	H	5.47160200	-1.14872200	-1.30289800	H	-4.79878200	-2.73076400	0.81653500
H	4.41617800	2.99815000	2.60056600	H	6.15138300	0.70203200	1.08921600	H	-4.20442800	-1.91801300	-2.10223900
H	6.23147800	3.04751300	2.33623600	H	7.14757000	0.47988000	-0.43512000	H	-5.76259500	-1.60896900	-1.18333900
H	6.54535100	1.41070800	4.24239900	H	7.38587400	-1.98656100	0.08455800	H	-6.06214700	-4.10408300	-0.86308100
H	4.72972600	1.37941200	4.50397300	H	6.39633200	-1.75476600	1.61144400	H	-4.50760500	-4.40256600	-1.78695000

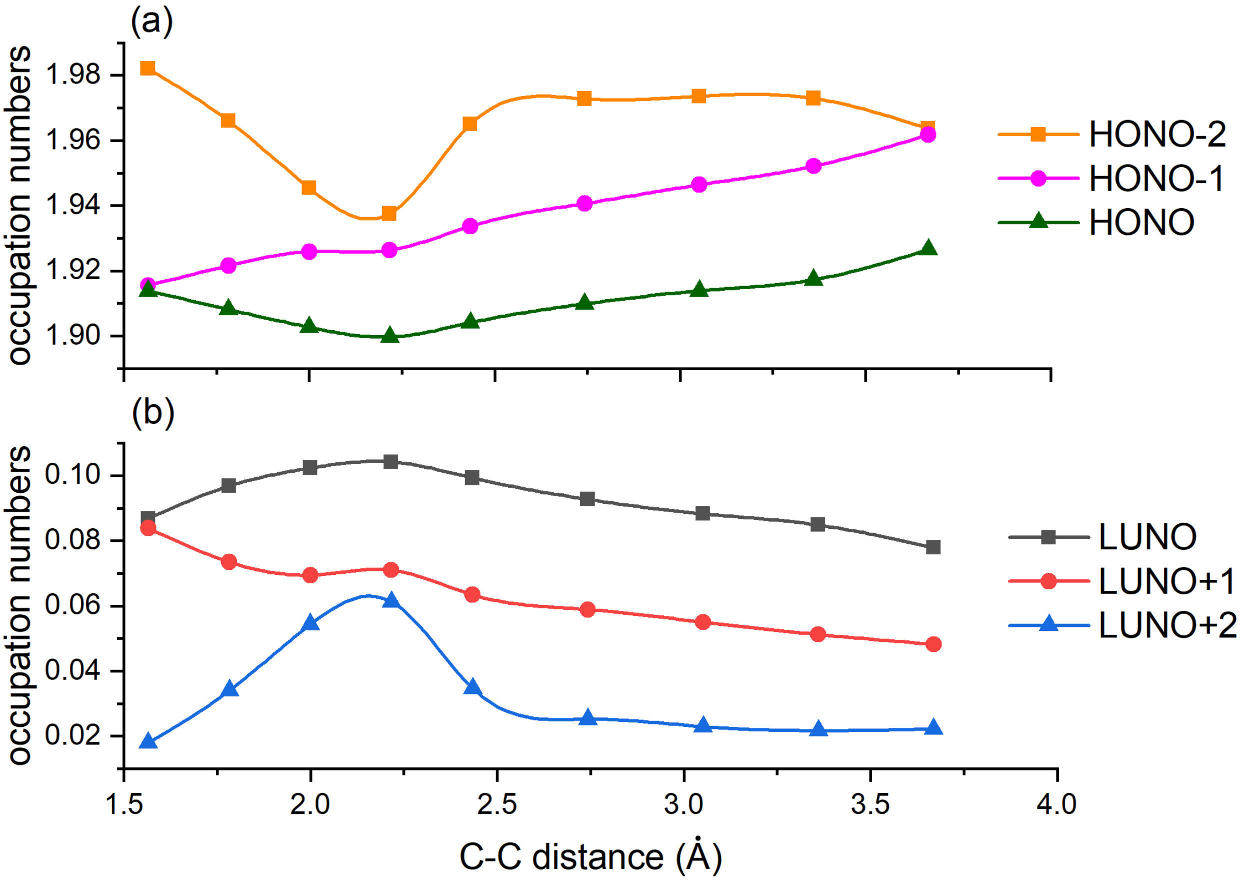

Figure S4. Occupation numbers of the natural orbitals versus the C–C distance, for the structures of the relaxed scan: (a) From HONO-2 to HONO orbitals; (b) From LUNO to LUNO+2 orbitals. The values have been computed at the CASSCF level with the cc-pVDZ(C)/sto-3G(H) basis set.
